# Manganese ion chelated nanoassemblies synergizing metalloimmunotherapy - chemodynamic for potentiating glioblastoma treatment

**DOI:** 10.1186/s12951-025-03845-6

**Published:** 2025-12-19

**Authors:** Yulei Mu, Zhen Zhang, Huiqun Zhou, Liang Ma, Bangheng Liu, Xu Hu, Chenjie Xu, Dong-An Wang

**Affiliations:** 1 Department of Biomedical Engineering, City University of Hong Kong, 83 Tat Chee Avenue, Kowloon, Hong Kong SAR China; 2 Center for Neuromusculoskeletal Restorative Medicine, InnoHK, HKSTP, Sha Tin, New Territories, 999077 Hong Kong SAR China; 3 Karolinska Institutet Ming Wai Lau Centre for Reparative Medicine, HKSTP, Sha Tin, 999077 Hong Kong SAR China; 4 Department of Biomedical Engineering, Chinese University of Hong Kong, Sha Tin, New Territories, 999077 Hong Kong SAR China

**Keywords:** Glioblastoma, Immunogenic cell death (ICD), Metalloimmunotherapy, Dendritic cell maturation, Tumor microenvironment reprogramming

## Abstract

Glioblastoma (GBM) remains among the most aggressive brain malignancies, necessitating the development of novel therapeutic strategies. In this study, we designed a multifunctional magnetic-targeting nanoassembly (MnBPDF) by chelating Mn²⁺ with a dopamine-modified albumin coat, encapsulating a magnetic core constructed with magnetic Fe₃O₄ and doxorubicin. MnBPDF effectively induces oxidative stress and triggers immunogenic cell death (ICD), thereby releasing damage-associated molecular patterns that prime antigen-presenting cells (APCs) and subsequently activate tumor-specific cytotoxic T lymphocyte (CTL) responses. Moreover, Mn²⁺ simultaneously enhances the activation of cyclic GMP-AMP synthase-stimulator of interferon genes (cGAS-STING) pathway, triggering robust type I interferon production and amplifying antitumor immune responses. By boosting antigen presentation by dendritic cells (DCs) in the GBM microenvironment, this system promotes CTL infiltration and inhibits tumor progression. The synergistic effects of magnetic targeting, ICD induction, and enhanced immune activation highlight MnBPDF as a highly effective therapeutic strategy for GBM.

**Graphical Abstract:**

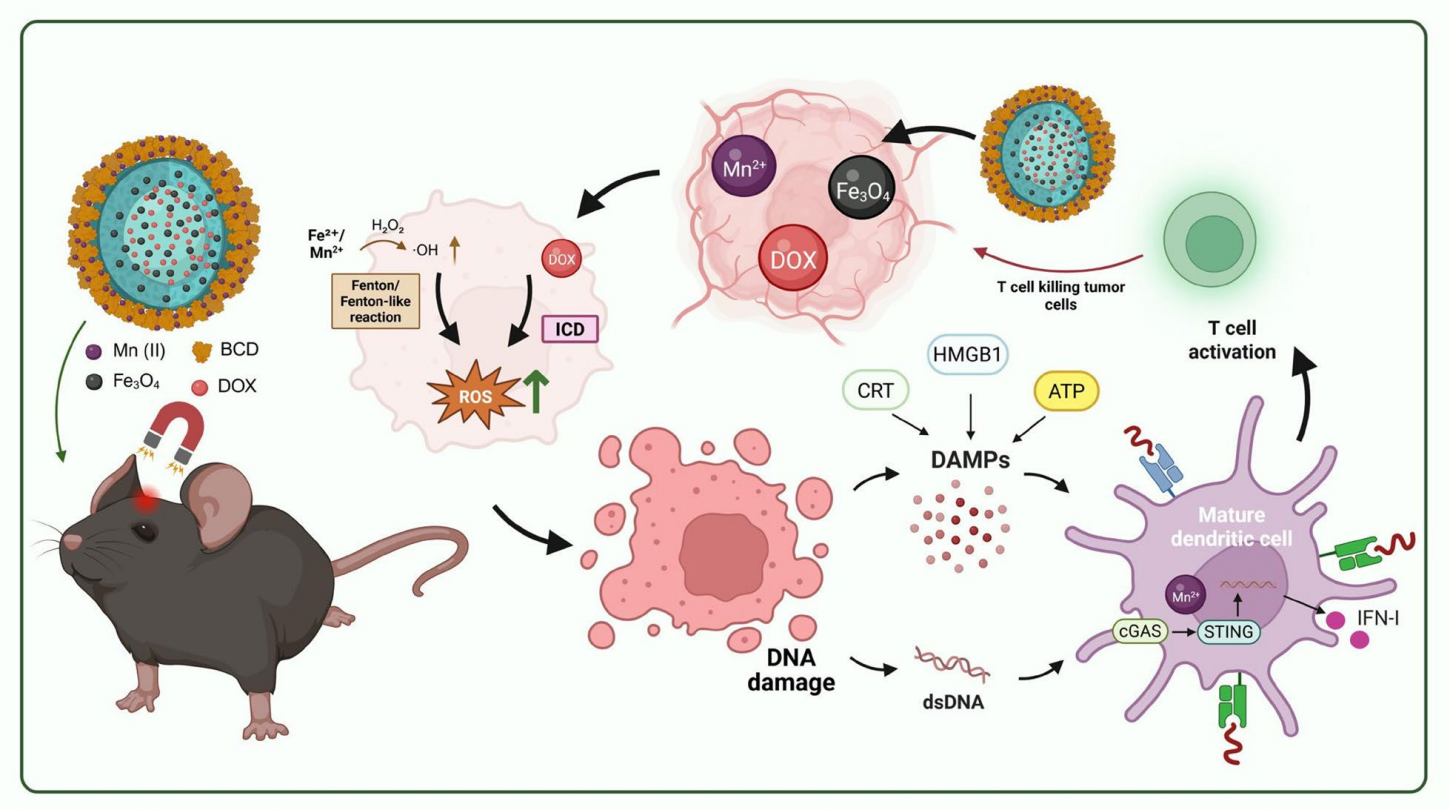

**Supplementary Information:**

The online version contains supplementary material available at 10.1186/s12951-025-03845-6.

## Introduction

Glioblastoma (GBM), classified as the most lethal primary intracranial malignancy, exhibits a median survival of only 12 to 18 months [1]. Current clinical approaches predominantly involve maximal surgical resection followed by temozolomide chemotherapy and radiotherapy [2]. Therapeutic efficacy remains severely limited by tumor heterogeneity and an immunosuppressive microenvironment that attenuates cytotoxic T lymphocyte (CTL) responses [3]. Emerging immunotherapies, such as cyclic GMP-AMP synthase-stimulator of interferon genes (cGAS-STING) pathway activation, immune checkpoint inhibition, and immunogenic cell death (ICD) induction, offer promising strategies for reprogramming the GBM microenvironment [4]. However, systemic delivery challenges and insufficient antigen presentation hamper clinical translation. Recent advances in nanomedicine offer multifunctional platforms for combinatorial chemo-immunotherapy through targeted delivery and microenvironment modulation [5–10].

Notably, metalloimmunotherapy, synergizing chemotherapeutics with STING agonists, has emerged as a paradigm-shifting strategy [11]. Manganese ions (Mn²⁺), an essential inorganic micronutrient critical for brain development and metabolism, traverse the blood-brain barrier as free ions, manganese-citrate complexes, and transferrin-bound Mn (Mn-Tf), undergoing central nervous system (CNS)-selective uptake regulated by robust homeostatic mechanisms under physiological exposure [12, 13]. Physiological Mn²⁺ concentrations range from 0.072 to 0.27 µM in systemic circulation and 20–53 µM in brain tissue [14, 15]. Recent advances highlight Mn²⁺ as potent cGAS co-activators that amplify STING signaling by stabilizing the cGAS-dsDNA complex, thereby enhancing the type I interferons (IFN-I) production [16, 17].

As a potent inducer of ICD, doxorubicin (DOX) has been extensively explored in GBM synergistic therapy through nanotechnology [18–23]. By inducing ICD, DOX facilitates damage-associated molecular patterns (DAMPs) release and leads to nuclear DNA damage and cytoplasmic leakage in tumor cells [24]. This process releases dsDNA, acting as a substrate for cyclic GMP-AMP synthesis. The presence of Mn²⁺ further enhances the STING pathway activation, thereby promoting the transcription of IFN-I. This cascade amplifies the antigen cross-presentation to CTLs, ultimately improving the cytotoxic activity of CTLs against the tumor [25, 26]. Moreover, DOX-induced apoptosis elevates tumor microenvironment (TME) reactive oxygen species (ROS) levels [27, 28], while Mn²⁺ enhances this effect through Fenton-like catalytic generation of hydroxyl radicals (·OH), synergistically amplifying oxidative stress to potentiate therapeutic efficacy [29, 30].

In the application of tissue engineering as a scaffold coating, dopamine-modified bovine serum albumin (BCD) can alleviate inflammatory and immune responses [31]. Through the pre-formation of albumin corona, the albumin coating can reduce non-specific interactions with blood components, thereby prolonging the blood circulation half-life of nanoparticles [32]. Modified by catechol, BCD can enhance the adhesion ability to nanoparticles and be used to chelate metal ions. In previous studies, it has been shown to have a good chelation ability for Fe³⁺and the ability to increase adhesion and biocompatibility [33].

**Fig. 1:**
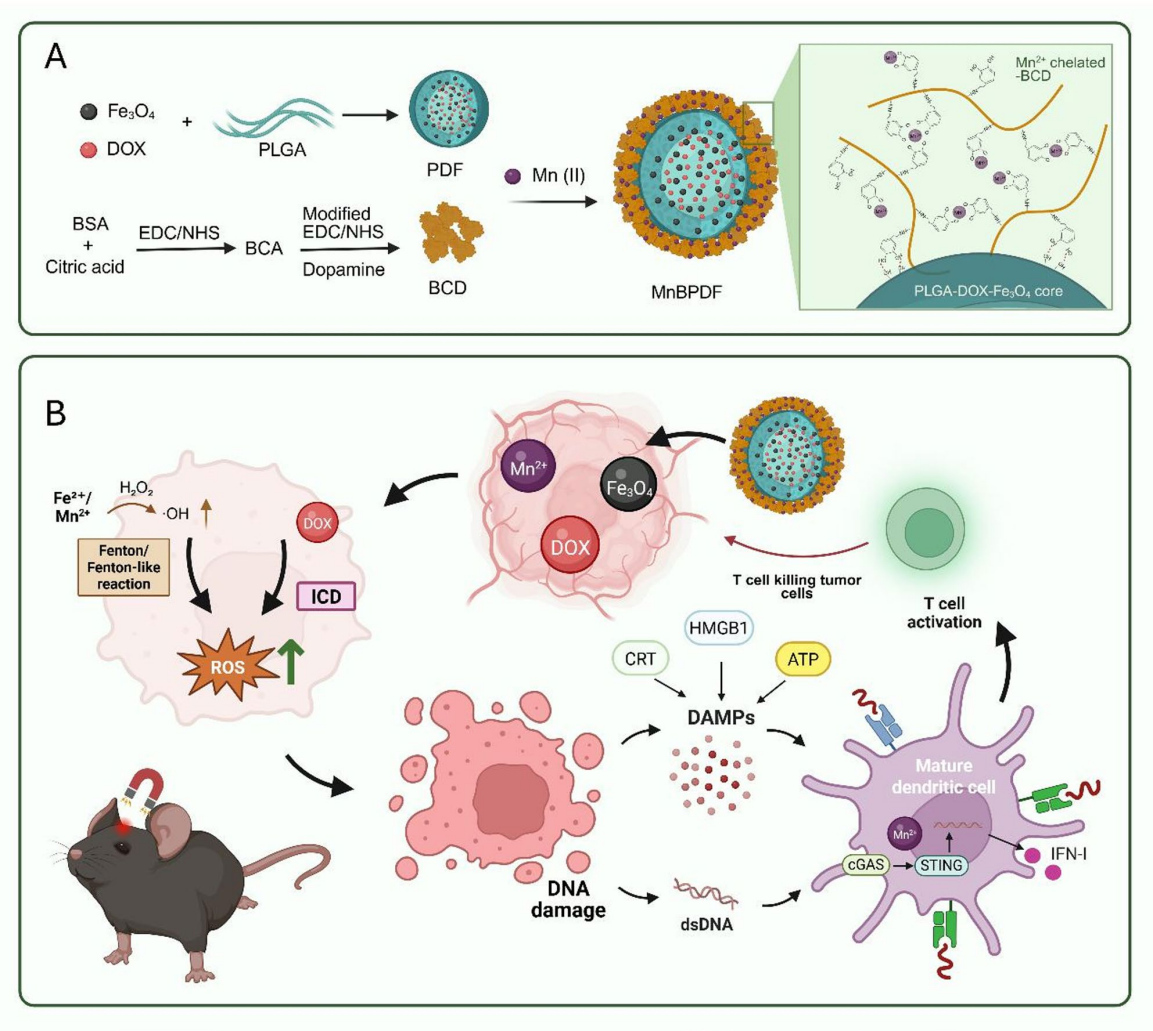
Schematic Illustration of MnBPDF Nanoassembly for GBM Therapy. (**A**) Structure of MnBPDF nanoassembly: BCD chelates Mn^2+^ and enhances adhesion by forming hydrogen bonds with the PLGA surface. (**B**) Following intravenous injection of MnBPDF into GBM-bearing mice, magnetic targeting facilitates accumulation in the TME, inducing ICD and tumor oxidative stress, thereby enhancing DCs maturation and antigen presentation, ultimately suppressing tumor progression

In this study, we developed a pH-sensitive magnetic nanoassembly, MnBPDF, for the treatment of GBM (Fig. 1). This system utilizes BCD to chelate Mn²⁺ as a coating and employs PLGA nanoparticles as the core, loaded with DOX and nano-Fe₃O₄, to achieve targeted and synergistic treatment of GBM. The acidic environment of the TME facilitates the dissociation of metal ion-catecholate complexes and the hydrolysis of PLGA, enabling the release of both Mn²⁺ and DOX [34]. DOX induces ICD, which, in combination with Mn²⁺, enhances the recognition of DNA released by tumor cells. This process activates the STING pathway, promoting dendritic cells (DCs) maturation, inducing IFN-I secretion, and increasing the infiltration and cytotoxic activity of CTLs in TME. Additionally, in the high ROS levels TME, Mn²⁺ along with Fe²⁺/Fe³⁺ from Fe₃O₄, can efficiently catalyze the conversion of overexpressed H₂O₂ into highly toxic ·OH via the Fenton reaction, which induces oxidative stress within tumor cells, leading to anti-tumor effect [35, 36]. This nanoassembly integrates immunotherapy, chemotherapy, and chemodynamic therapy into a synergistic platform, enabling a multifaceted therapeutic strategy against GBM. Through the concurrent induction of ICD, activation of the STING signaling pathway, and amplification of oxidative stress via Fenton reactions, this system offers a comprehensive and coordinated mechanism of action that surpasses traditional magnetically guided approaches, thereby enhancing therapeutic efficacy.

## Materials and methods

### Materials and cell lines

Unless otherwise stated, all chemical reagents were sourced from Sigma-Aldrich, and cell culture consumables were procured from Thermo Fisher Scientific. The GL261, GL261-Luc, and DC2.4 cell lines were acquired from Ubigene (China), while the U251 cell lines were obtained from Procell (China). All cell lines underwent proper authentication via short tandem repeat (STR) profiling and were maintained under standard culture conditions (37 °C, 5% CO₂). The DC2.4 cells were cultured in RPMI-1640 medium supplemented with 10% FBS (fetal bovine serum), while other cell lines were maintained in Dulbecco’s Modified Eagle Medium containing 10% FBS and 1% penicillin-streptomycin.

### Preparation and characterization of MnBPDF

The magnetic-chemotherapeutic core PDF was prepared via water-oil-water double emulsion. Initially, 10 nm Fe₃O₄ nanoparticles (200 µL, 5 mg/mL) and 1 mL DOX·HCl (10 mg/mL in deionized water) were homogenized using a vortex mixer. The mixture was then injected into 10 mL of acetone containing 100 mg 0.67 dL/g acid-terminated 50:50 poly(lactic-co-glycolic acid) (PLGA, Aladdin) using a syringe pump at 0.1 mL/min under mechanical stirring. The primary emulsion was subsequently added into 20 mL of 4% (w/v) polyvinyl alcohol (PVA) aqueous solution, which served as an emulsifier to stabilize the emulsion system. The mixture was then sonicated in an ice bath for three cycles (30 s pulse, 15 s interval), followed by continuous stirring for 12 h at 25 °C to allow for acetone evaporation. Centrifugation at 10,000 ×g with three washing cycles using deionized water at 4 ℃.

BCD was synthesized by modifying the previous methodology [37]: BSA (bovine serum albumin) (0.3 g/mL in PBS, pH 5.5) was reacted with 50 mM citric acid, 30 mM EDC, and 30 mM NHS for 6 h (hr) at 37 °C. The resulting BCA was dialyzed and lyophilized. Subsequently, 1 g of BCA reacted with 1 g of dopamine hydrochloride using 0.5 g EDC/0.5 g NHS under argon (Ar) protection at 37 °C for 8 h at pH 6 in 100 mL PBS. The final product, BCD, was dialyzed, lyophilized, and stored at 4 °C.

The nanoassembly MnBPDF was constructed through coordinated self-assembly. PDF nanoparticles (1 mg) and BCD (2 mg) were mixed in 2 mL PBS (pH 7.5) and incubated at 4℃ for 12 h. 200 µL of 20 mg/mL MnCl₂·4 H₂O was added dropwise followed by orbital shaking (200 rpm, 12 h, 4 °C). Centrifugal washing (10,000 ×g ×3) with deionized water, stored at 4 °C.

Nanoparticle size distribution and surface charge (Zeta potential) were analyzed by dynamic light scattering (DLS, Malvern Zetasizer ZEN3600). Polydispersity index (PDI) values were calculated and analyzed using ZS Xplorer software. Transmission electron microscopy (TEM, FEI Talos F200X) and environmental scanning electron microscopy (SEM, FEI Quanta 250) were utilized to characterize the morphology of MnBPDF. Lyophilized BCD, PDF and MnBPDF powders were subjected to crystallographic analysis using X-ray Diffraction (XRD, Bruker D8 Venture). Magnetic hysteresis loops were acquired using Vibrating Sample Magnetometry (VSM, Lake Shore). X-ray photoelectron spectroscopy (XPS, Thermo Scientific K-Alpha) was employed to analyze the elemental composition and surface chemical states of lyophilized MnBPDF nanoparticles. DOX loading capacity (LC) and encapsulation efficiency (EE) were quantified using UV-Vis spectroscopy (Molecular Devices SpectraMax M3) at λ = 480 nm.

$$\:LC\left(\%\right)=\frac{{Drug}_{loaded}}{NPs\:mass}\times\:100\%$$

$$\:EE\:\left(\%\right)=(1-\frac{{Drug}_{free}\:}{{Drug}_{total}})\times\:100\%$$

Drug release kinetics were evaluated via dynamic dialysis (MWCO: 2 kDa). MnBPDF were suspended in 15 mL of PBS (pH 5.5 or 7.4) at a concentration of 0.1 mg/mL and incubated under constant agitation (100 rpm) at 37 °C. 1 mL solution was collected at predetermined intervals with equal volume pre-warmed PBS replenishment. The concentration of Mn²⁺ in MnBPDF was quantified by ICP-OES (Agilent 720ES). The synthesis of BCD was confirmed through ¹H nuclear magnetic resonance (Bruker Plus 600 MHz).

### Cellular uptake of MnBPDF

GL261 glioma cells were cultured in confocal dishes, treated with DOX, PDF, and MnBPDF at a concentration of 0.5 µM DOX, and incubated for 4 h. Confocal laser scanning microscopy (CLSM) characterized cellular uptake of the drugs by GL261 cells.

### Mitochondrial membrane potential assessment

GL261 cells were cultured in confocal dishes and treated with either drugs containing 0.5 µM DOX or PBS for 24 h. Following treatment, cells were incubated with 25 mM JC-1 dye (Beyotime) for 30 min at 37 °C. CLSM quantitatively analyzed mitochondrial polarization states after PBS washing.

### Measurement of intracellular ROS levels

GL261 cells were treated with either drug (containing 0.5 µM DOX) or PBS for 24 h and stained with 10 µM DCFH-DA for 20 min. A flow cytometer (BD FACSVerse™) was utilized to characterize the ROS level. Data was analyzed using FlowJo v10.8 software, with mean fluorescence intensity (MFI) reported in arbitrary units (a.u.).

### Glutathione peroxidase (GPx) activity assay

Following 24 h treatment with either drug containing 0.5 µM DOX or PBS in 6-well plates, GL261 cells were harvested and lysed. Lysates were centrifuged at 12,000 × g, and supernatants were collected for GPx activity measurement.

### Lipid peroxidation assessment

GL261 glioma cells were cultured in confocal dishes and treated with either drug containing 0.5 µM DOX or PBS for 24 h. After treatment, cells were incubated with BODIPY™ 581/591 C11 dye (Thermo Fisher Scientific) for 30 min at 37 °C. Following PBS washing, lipid peroxidation levels were evaluated using CLSM. The oxidation-induced shift in fluorescence was quantitatively analyzed using ImageJ.

### Establishment of the orthotopic intracranial GBM model

C57BL/6 mice (6–8 weeks) were anesthetized and secured in a rotational digital stereotaxic frame (RWD Life Science) with mouse ear bars and tooth hook. Scalp incision (5 mm sagittal) followed by cranial window creation (1 mm diameter) using micro-drill at the coordinates relative to bregma (ML = −1.5 mm, AP = −1 mm, DV = −3.5 mm). GL261-Luc cells (5 × 10⁴ in 4 µL PBS) were injected via a 33G Hamilton syringe at 1 µL/min.

### In vivo evaluation of brain tumor progression

Mice tumor progression was monitored utilizing In Vivo Imaging System (IVIS, Perkin Elmer) Spectrum. Mice were administered an intraperitoneal injection of a D-Luciferin potassium salt (15 mg/mL, Beyotime) at 10 µL/g body weight for bioluminescence imaging.

### In vivo brain targeting ability of MnBPDF

To investigate the brain targeting capability of MnBPDF, Cy5-labeled MnBPD (MnBPDF without Fe_3_O_4_) and MnBPDF were separately administered as a single dose (250 µL) on day 10 post-tumor inoculation to evaluate their GBM targeting capabilities. Cylindrical NdFeB magnet (N52, Φ10 × 5 mm) fixed 2 mm above the mice skull for magnetic intervention. The distribution of nanoparticles was assessed using the IVIS. The fluorescence intensity in organs was measured to study the distribution across different tissues.

### Calreticulin (CRT) surface exposure analysis

GL261 cells were treated with PD (PDF without Fe_3_O_4_), PDF, MnBPD, and MnBPDF for 24 h (each group containing 0.5 µM DOX). To assess the release of CRT, cells were stained with Anti-Calreticulin antibody (Ab 92516) overnight at 4 °C. Goat anti-rabbit secondary antibody (Ab150077) were stained for 30 min. CLSM was used for imaging. All primary antibodies were sourced from Abcam.

### Extracellular HMGB1 quantification

For the quantification of extracellular high-mobility group box 1 (HMGB1), GL261 cell supernatants collected 24 h post-treatment were subjected to centrifugation (12000×g, 5 min, 4 °C) to collect the supernatants for ELISA assays (FineTest) to determine the levels of HMGB1 release.

### ATP release profiling

In profiling adenosine triphosphate (ATP) release, GL261 cell supernatants were collected 24 h post-treatment. The extracellular ATP levels were determined utilizing an ATP assay kit (Beyotime) based on the firefly luciferase method. Luminescence intensity (RLU) was measured using the IVIS Spectrum.

### In vitro DCs maturation study

To evaluate the capacity of MnBPDF to promote DCs maturation through tumor-derived immunogenic signals, GL261 cells were treated with PBS, PDF, MnBPD, and MnBPDF for 48 h. The supernatants containing tumor cell-derived immunogenic factors were collected for DCs activation.

DC2.4 cells were cultured in 12-well plates and exposed to 1 mL of the collected supernatant for 24 h. Post-treatment, DCs were harvested and immunophenotyped via flow cytometry using fluorophore-conjugated antibodies: PE-CD11c (117308), APC-CD86 (105012), and FITC-CD80 (104706). All flow cytometric antibodies were sourced from BioLegend.

DC2.4 cells were exposed to conditioned media for 24 h, followed by multi-omics characterization employing 4D-DIA proteomic profiling (Bruker timsTOF HT) and complemented by RNA sequencing (Illumina).

### In vivo therapeutic intervention

GL261 tumor-bearing mice received 250 µL systemic administrations of DOX, PD, MnBPD, and MnBPDF containing 2 mg/kg of DOX via intravenous tail vein injection. Dosing occurred post-inoculation on days 8, 10, 12, 15, 17, and 19. Magnetic targeting groups received continuous NdFeB magnet application over the cranial region. Tumor progression was quantified through bioluminescent imaging by IVIS Spectrum.

### Immune cells analysis in brain tissue

Single-cell suspensions from brain tumors (day 21) were enzymatically dissociated and immune-stained for flow cytometry. The suspensions were incubated with CD11c-PE (117308), CD80-FITC (104706), CD86-APC (105012), and I-A/I-E-PerCP (107624) for DCs staining; CD3-APC (100236), CD4-PE (100408), CD8b.2-FITC (140404) for T-cell staining. All flow cytometric antibodies were sourced from BioLegend.

### Mouse brain tissues immunohistochemical (IHC) staining

Paraffin sections of mouse brain tissues were prepared and subjected to IHC staining. To assess tumor growth and proliferation, sections were incubated with Anti-VEGFR2 (ab2349) or Anti-Ki67 antibody (ab15580). Anti-CD86 antibody (ab234000) was used to evaluate DCs maturity, while Anti-CD3 epsilon antibody (ab16669) and Anti-CD8 alpha antibody (ab217344) were employed to detect T cell infiltration into the tumor. Additionally, anti-CRT (ab92516) and anti-HMGB1 (ab79823) antibodies were used to investigate the release of DAMPs. The primary antibodies were allowed to incubate overnight at 4 °C. After PBS washing, the sections were incubated with secondary antibodies (Proteintech, PK10006) and subjected to DAB staining.

### TUNEL staining

TUNEL staining using the TUNEL assay kit (Beyotime) was performed on paraffin sections of mouse brain tumors, followed by CLSM imaging to analyze apoptosis in tumor cells.

### Animal maintenance and ethical statement

All experiments complied with the Animal Research Ethics Sub-Committee (AEC, A-0828) approval to ensure that the experiments met ethical guidelines and standards.

### Statistical analysis

GraphPad Prism 10.1.2 was utilized for analysis with one-way ANOVA unless otherwise stated. Data represent mean ± SD with significance thresholds: *p < 0.05, **p < 0.01, ***p < 0.001, ****p < 0.0001; ns (nonsignificant).

## Results and discussion

### Synthesis and characterization of MnBPDF nanoassembly

**Fig. 2:**
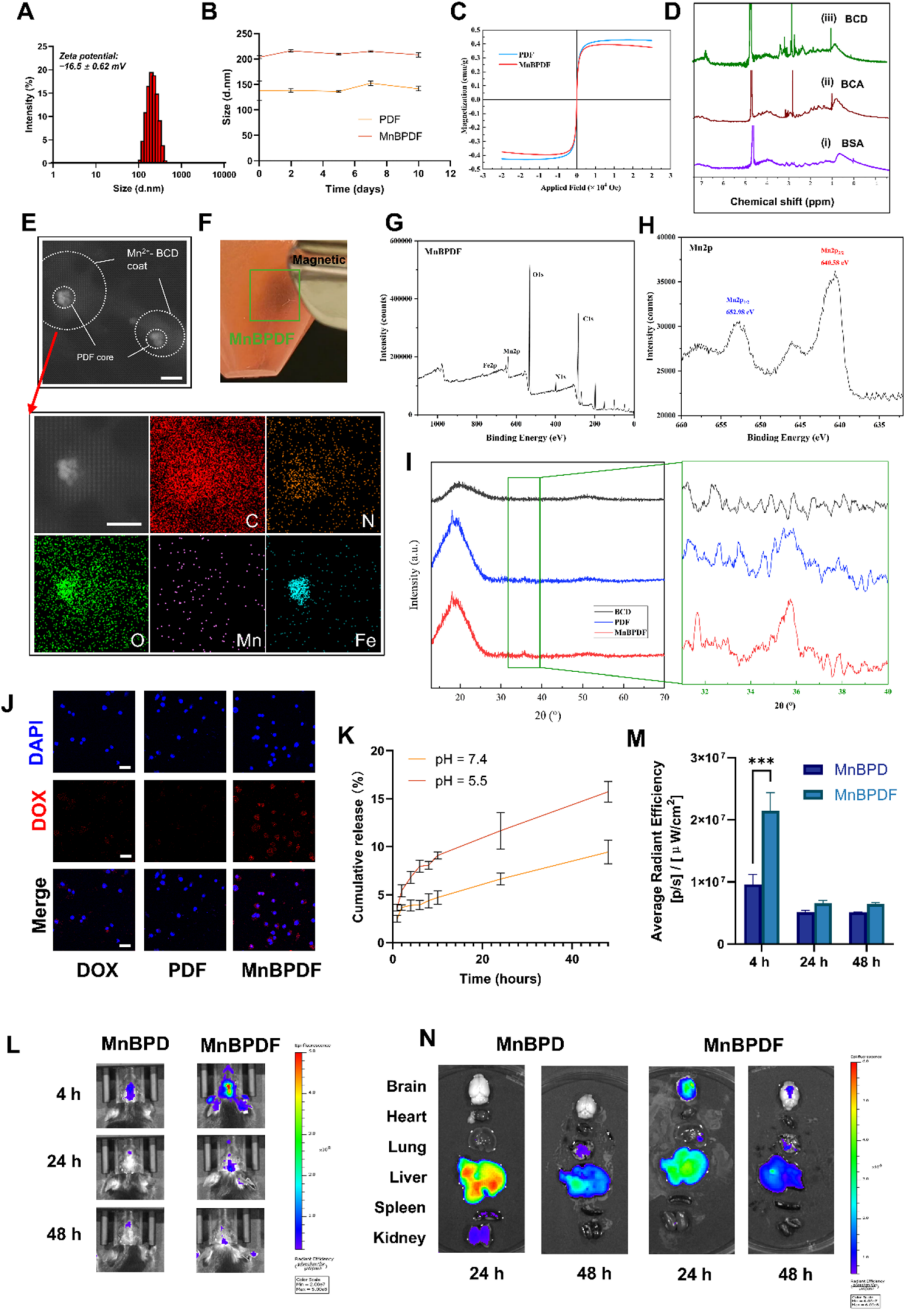
Synthesis and Functional Characterization of MnBPDF Nanoassembly. (**A**) Hydrodynamic diameter distribution of MnBPDF measured by DLS. (**B**) Hydrodynamic diameter stability of PDF and MnBPDF for 4 ℃ 10 days. (**C**) VSM curves of PDF and MnBPDF. (**D**) ¹H NMR spectra validating BCD synthesis: (i) native BSA, (ii) citric acid-grafted BSA (BCA), (iii) dopamine-functionalized BCA (BCD). (**E**) TEM and the corresponding EDS elemental mapping images of MnBPDF. Scale bar = 50 nm. (**F**) Magnetic responsiveness of MnBPDF under external NdFeB magnet. (**G**) XPS of MnBPDF. (**H**) High-resolution Mn 2p spectrum of MnBPDF. (**I**) XRD pattern of BCD, MnBPD and MnBPDF. (**J**) CLSM images of GL261 cells after 4 h treatment with PDF or MnBPDF (red: DOX, blue: nuclei). Scale bar = 25 μm. (**K**) DOX release profiles from MnBPDF in PBS (pH 5.5 vs. 7.4) over 48 h. (*n* = 3). (**L**) In vivo brain accumulation of Cy5-labeled MnBPD and MnBPDF characterized by IVIS at 4 h, 24 h, 48 h. (**M**) Semiquantitative analysis of brain fluorescence intensity at 4 h, 24 h, 48 h post-injection (mean ± SEM, ***p < 0.001). (**N**) Ex vivo biodistribution of MnBPD and MnBPDF in major organs at 24 h and 48 h

MnBPDF nanoassemblies were synthesized through a sequential fabrication process (Fig. 1A). The magnetic-chemotherapeutic core (PDF) was synthesized via a modified water-oil-water double emulsion, encapsulating Fe₃O₄ nanoparticles (10 nm) and DOX within PLGA. Initial characterization of the PDF nanoparticles revealed a hydrodynamic diameter of 137.7 ± 19.74 nm and a polydispersity index (PDI) of 0.381 ± 0.006 (Fig. S1). BCD was pre-adhered onto the PDF surface, forming the intermediate BPDF. This surface modification increased the hydrodynamic diameter to 236.3 ± 19.07 nm while slightly reducing PDI to 0.345 ± 0.015. MnBPDF nanoassembly was achieved through Mn²⁺ mediated coordination of catechol groups on the BCD coating. This chelation-driven structural consolidation significantly improved colloidal stability, yielding monodisperse nanoparticles with a refined hydrodynamic diameter of 203.5 ± 3.5 nm and a remarkably low PDI of 0.072 ± 0.005 (Fig. 2A). Zeta potential measurements of MnBPDF confirmed a negatively charged surface (− 16.5 ± 0.62 mV). Long-term colloidal stability analysis revealed that MnBPDF maintained hydrodynamic diameter profiles over 10-day storage at 4 °C (Fig. 2B).

TEM (Fig. 2E) and SEM (Fig. S2) further characterized the morphology of MnBPDF. EDS mapping of MnBPDF showed a core shell structure. ¹H NMR spectroscopy confirmed the successful synthesis of BCD, as evidenced by emerging peaks corresponding to methylene groups in CA (δ2.6–2.8 ppm), aromatic protons (δ 6.6–6.8 ppm), and ethylamine chain protons (δ3.0–3.3 ppm) (Fig. 2D) [38, 39]. The FTIR spectra confirmed the successful integration of PLGA, DOX, and BCD in MnBPDF, as evidenced by characteristic ester (C = O), aromatic (C = C), and amide (I and II) absorption bands (Fig. S4). XPS spectrum of MnBPDF revealed characteristic peaks at 283.9 eV (C 1 s), 399.0 eV (N 1 s), 531.5 eV (O 1 s), 640.7 eV (Mn 2p), and 721.3 eV (Fe 2p), confirming the presence of key elements within the nanoassemblies (Fig. 2G). The relatively weak Fe 2p signal is attributed to the encapsulation of Fe₃O₄ within the nanoassemblies, which attenuates surface detection. High-resolution Mn 2p spectrum exhibited peaks at 640.6 eV (Mn 2p_3/2_) and 653.0 eV (Mn 2p_1/2_), consistent with Mn²⁺, confirming the successful chelation of Mn²⁺ onto the BCD coating (Fig. 2H). The crystalline architecture of MnBPDF was systematically validated through XRD analysis. The broadened diffraction hump at 20° arose from the amorphous superposition of PLGA polymer and BCD shells and characteristic peaks at 35.5° confirming the Fe₃O₄ cores (Fig. 2I). MnBPDF exhibited robust magnetic responsiveness, with rapid accumulation under a NdFeB magnet (Fig. 2F). The VSM curves further illustrate the superparamagnetism of PDF and MnBPDF, with saturation magnetization values of 0.43 and 0.40 emu/g (Fig. 2C). This indicates that the magnetization strength of PDF remains largely unchanged after the addition of the BCD-Mn²⁺ coating [40]. UV-vis characterized the LC and EE of DOX in MnBPDF (LC = 2.8%, EE = 35.7%). ICP-OES analysis revealed a Mn²⁺ concentration of 89.7 ± 0.5 µg/mL in 2 mg/mL MnBPDF (corresponding to 4.5 wt% loading efficiency), while remaining below the established murine safety threshold for Mn²⁺ administration [41]. Cumulative release of DOX in MnBPDF demonstrated accelerated DOX release under acidic conditions (pH 5.5: 15.72 ± 1.07% at 48 h vs. pH 7.4: 9.44 ± 1.22%), aligning with the GBM TME (Fig. 2K).

In vitro studies on cellular uptake revealed increased DOX fluorescence in GL261 glioma cells treated with MnBPDF compared to free DOX and PDF (Fig. 2J), attributed to enhanced endocytosis facilitated by the Mn²⁺-chelated BCD coating. To evaluate the brain-targeting ability of MnBPDF, 250 µL of Cy5-labeled MnBPDF was intravenously injected into C57BL/6 mice. IVIS imaging of the mouse brain fluorescence revealed a significant signal in the mouse brain at 4 h post-injection when an external magnetic field was applied over the brain, indicating effective accumulation of MnBPDF (Fig. 2L). The quantitative analysis of the fluorescence signal showed that at 4 h, the fluorescence signal in the MnBPDF group was significantly higher (2.2-fold) than the MnBPD group without Fe_3_O_4_ addition (Fig. 2M). Ex vivo organ IVIS fluorescence imaging indicated that MnBPD tended to accumulate more in the liver, while MnBPDF showed higher accumulation in the mouse brain. By 48 h, most of the nanoassemblies had been metabolized (Fig. 2N).

### MnBPDF induce oxidative stress and ICD in vitro

**Fig. 3:**
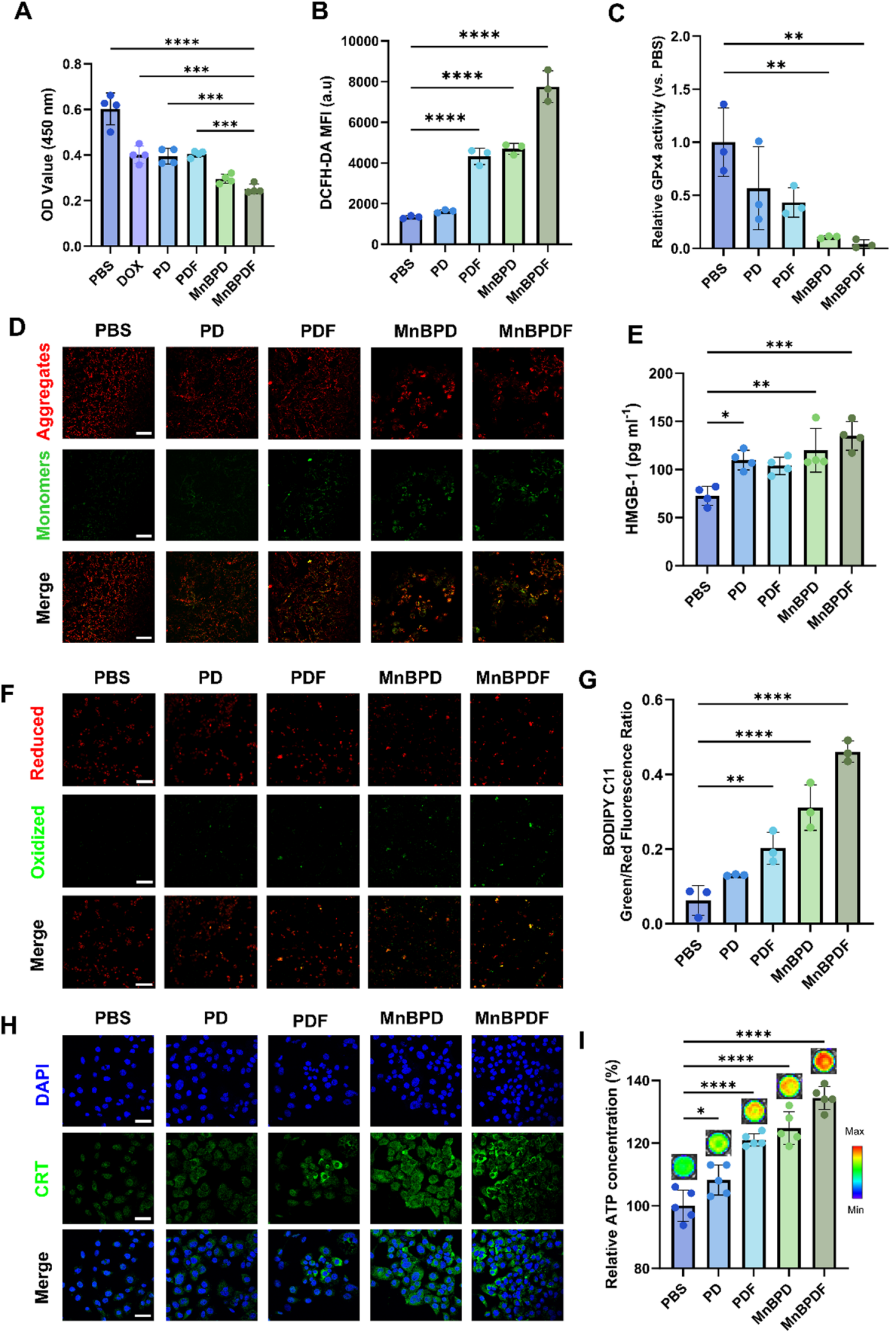
MnBPDF Induces ICD and Oxidative Stress In Vitro. (**A**) Cell viability of GL261 cells with different treatment groups for 48 h (*n* = 4). (**B**) Intracellular ROS levels were quantified by DCFH-DA fluorescence using flow cytometry (*n* = 3). (**C**) GPx4 activity in GL261 cells normalized to PBS control (*n* = 3). (**D**) CLSM images of JC-1 staining showing mitochondrial depolarization. Scale bar = 50 μm. (**E**) HMGB1 secretion in the supernatant was quantified by ELISA (*n* = 4). (**F**) CLSM images of BODIPY™ 581/591 C11 staining for lipid peroxidation in GL261 cells. Scale bar = 100 μm. (**G**) Quantification of BODIPY™ C11 fluorescence intensity (*n* = 3). (**H**) CLSM image of CRT exposure on GL261 cell membranes. Scale bar = 50 μm. (**I**) ATP release quantified via luciferase-based bioluminescence assay detected by IVIS (*n* = 5)

CCK-8 analysis revealed a significant reduction in GL261 cell viability following 48 h treatment with MnBPD and MnBPDF compared to DOX, PD, and PDF groups (Fig. 3A), indicating a synergistic effect of DOX and Mn^2+^ in inhibiting GL261 cell proliferation.

Under the acid TME, protonation-induced ligand dissociation released Mn²⁺ ions from BCD while simultaneously triggering PLGA hydrolysis to expose Fe₃O₄ nanoparticles and release DOX. In the ROS-enriched TME, the liberated Mn²⁺ synergized with Fe₃O₄ to establish a self-reinforcing catalytic cycle. Flow cytometry revealed a 5.8-fold increase in intracellular ROS levels (Fig. 3B), while GPx4 activity showed a 96% depletion relative to controls (Fig. 3C) after 24 h treatment, confirming systemic oxidative stress. Mitochondrial involvement was substantiated by JC-1 staining, showing pronounced depolarization of mitochondrial membrane potential in MnBPDF-treated cells (Fig. 3D). Lipid peroxidation levels were assessed using BODIPY™ 581/591 C11. CLSM revealed a marked increase in green fluorescence intensity in the MnBPDF group, indicative of elevated lipid peroxidation (Fig. 3F). Quantitative analysis demonstrated a significant increase in the green/red fluorescence ratio compared to other treatment groups (Fig. 3G), further confirming lipid peroxidation–mediated membrane damage associated with ferroptosis.

To evaluate the ICD-inducing capacity of MnBPDF, we systematically analyzed the release of DAMPs, including CRT, HMGB1, and ATP. CLSM revealed pronounced CRT translocation to the cell membrane after treating with MnBPDF for 24 h (Fig. 3H). ELISA quantification revealed a 1.9-fold increase in extracellular HMGB1 levels in MnBPDF-treated supernatants versus PBS (Fig. 3E), confirming nuclear DAMPs release. Concurrently, ATP release measured by luciferase assay revealed a 1.3-fold increase in MnBPDF-treated groups (Fig. 3I), collectively validating robust ICD activation.

### MnBPDF enhances antigen-presenting capacity of DCs in vitro

**Fig. 4:**
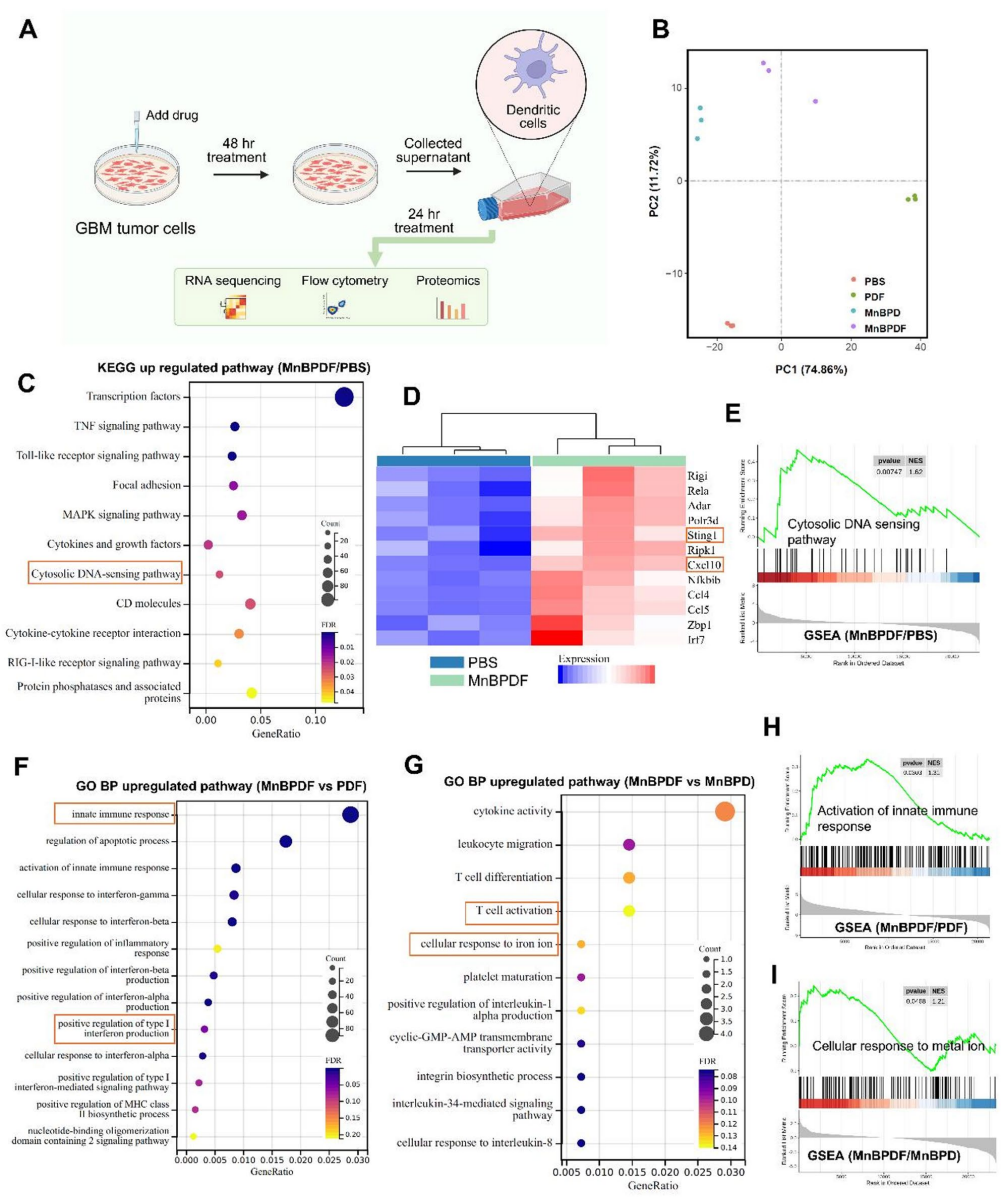
RNA Sequencing Reveals MnBPDF-Induced DCs Maturation and Pro-Antitumor Immune Activation. (**A**) Schematic of in vitro experimental workflow for assessing DCs maturation under treatment. (**B**) PCA of RNA sequencing result (*n* = 3). (**C)** KEGG pathway enrichment analysis of upregulated pathways in MnBPDF vs. PBS controls (FDR < 0.05). (**D**) Heatmap of differentially expressed genes in the cytosolic DNA-sensing pathway, z-score-normalized. (**E)** GSEA validating activation of cytosolic DNA-sensing pathway in MnBPDF vs. PBS. (**F**) GO BP enrichment of upregulated pathways in MnBPDF vs. PDF (*p* < 0.05). (**G**) GO BP enrichment of upregulated pathways in MnBPDF vs. MnBPD (*p* < 0.05). (**H**) GSEA of GO BP activation of innate immune response pathway in MnBPDF vs. PDF group. (**I)** GSEA of GO BP cellular response to metal ion pathway. MnBPDF vs. MnBPD group

To validate that MnBPDF enhances DCs mediated tumor antigen presentation through induction of ICD and Mn²⁺-augmented cGAS-STING pathway activation [42, 43], we assessed DC maturation in vitro. DC2.4 cells were exposed to supernatants collected from GL261 cells treated with PBS, PD, PDF, MnBPD, or MnBPDF for 24 h (Fig. 4A). Transcriptomic profiling revealed MnBPDF-induced activation of DCs-mediated anti-tumor immunity. Principal component analysis (PCA) demonstrated significant intergroup variance in global gene expression profiles (Fig. 4B). Pathway enrichment analysis identified pronounced upregulation of pro-immunogenic processes in the MnBPDF group, as evidenced by KEGG pathway analysis showing significant enrichment in tumoricidal immune activation pathways compared to PBS controls (Fig. 4C). Mechanistically, Gene Set Enrichment Analysis (GSEA) confirmed robust activation of the cytosolic DNA-sensing pathway in MnBPDF group (Fig. 4E), which was further corroborated by coordinated upregulation of hallmark genes including CXCL10 and STING1 in pathway-specific clustering heatmaps (Fig. 4D). These transcriptional signatures collectively indicate MnBPDF-mediated potentiation of the cGAS-STING signaling axis and antitumor immune response.

Compared with the PDF group, the MnBPDF group exhibited increased expression of IFN-I production and IFN-I-mediated signaling pathways, as shown by GO BP (Biological Process) enrichment (Fig. 4F), along with GSEA demonstrating upregulation of the activation of innate immune response pathway (Fig. 4H). These findings suggest that the inclusion of BCD-Mn²⁺ significantly boosts the antitumor immune response. Furthermore, compared to the MnBPD group, MnBPDF treatment led to upregulation of pathways associated with T cell activation and cellular response to iron ions, as revealed by GO BP analysis (Fig. 4G). GSEA further showed enrichment of the cellular response to metal ions pathway (Fig. 4I), indicating that the combined presence of Mn²⁺ and Fe₃O₄ in the MnBPDF formulation synergistically enhances T cell activation, cytokine secretion, and cellular responsiveness to metal ions within the immune microenvironment.

**Fig. 5:**
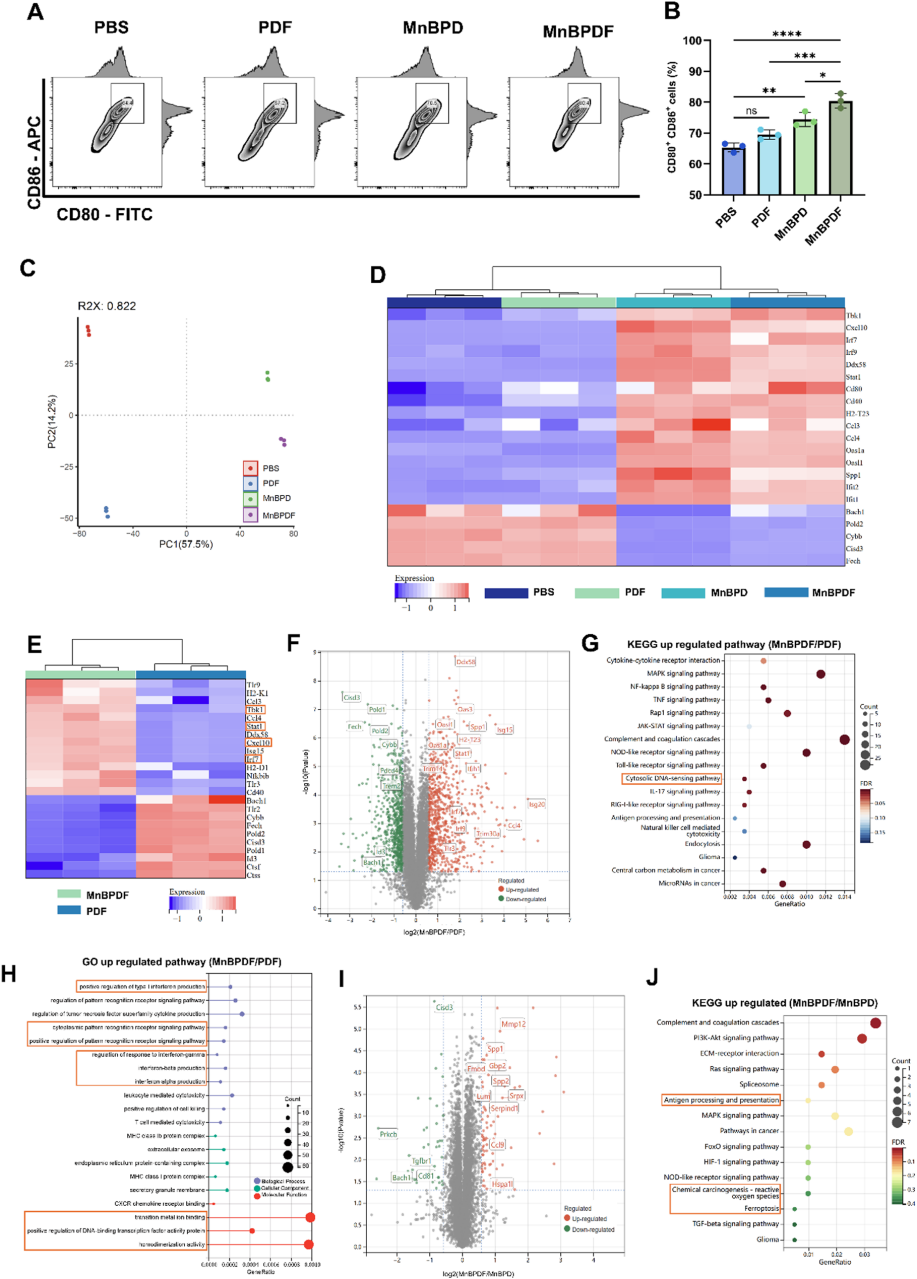
Protein Expression Confirms MnBPDF-Specific Activation of Immune and Oxidative Stress Pathways. (**A**) Flow cytometry profiles of DCs maturation markers (CD80/CD86) on DC2.4 cells cultured with conditioned media from GL261 cells treated with PBS, PDF, MnBPD, or MnBPDF for 24 h. (**B**) Quantitative analysis of CD80⁺CD86⁺ mature dendritic DCs. (*n* = 3). (**C**) PCA of global proteomes demonstrates distinct clustering among treatment groups. (**D**) Heatmap of hierarchical clustering based on Euclidean distance of z-score-normalized intensity. (|fold change| >1.5, *p* < 0.05). (**E**) Heatmap of hierarchical clustering based on Euclidean distance of z-score-normalized intensity between MnBPDF and PDF groups. (**F**) Volcano plot analysis between MnBPDF and PDF group (|fold change| >1.5, *p* < 0.05). (**G**) KEGG pathway enrichment analysis of upregulated pathways between MnBPDF group and PDF group. (FDR < 0.05, *p* < 0.05). (**H**) GO enrichment analysis of biological processes upregulated between MnBPDF and PDF groups (FDR < 0.05, *p* < 0.05). (**I**) Volcano plot analysis between MnBPDF and MnBPD group (|fold change| >1.5, *p* < 0.05). (**J)** KEGG pathway enrichment analysis of upregulated pathways between MnBPDF and MnBPD groups (FDR < 0.05, *p* < 0.05)

Flow cytometry revealed that the MnBPDF group induced significantly higher DC maturation, with 80.43 ± 2.35% CD80⁺CD86⁺ mature DCs, whereas the PBS group exhibited 65.33% ± 1.46% (Fig. 5A and B). Proteomic profiling further corroborated the enhanced anti-tumor immune responses elicited by MnBPDF. PCA of global proteomic profiles demonstrated distinct clustering among treatment groups (Fig. 5C), with MnBPD- and MnBPDF-treated groups exhibiting significant enrichment of proteins associated with antigen presentation (e.g., CD80), interferon signaling (e.g., STAT1, ISG15), and STING pathway activation (e.g., TBK1, IRF7, CXCL10) compared to controls (Fig. 5D). Comparative proteomic profiling of MnBPDF versus PDF groups revealed marked differences in protein expression patterns. Heatmap and volcano plot analyses (Fig. 5E and F) identified pronounced upregulation of immune-related proteins in the MnBPDF group. KEGG pathway enrichment analysis confirmed significant activation of the cytosolic DNA-sensing pathway and antigen processing/presentation pathways in MnBPDF groups compared to PDF groups (Fig. 5G), indicating activation of the cGAS-STING pathway. Gene Ontology (GO) term analysis further validated enhanced IFN-I production and MHC I-mediated immune responses (Fig. 5H). Comparative proteomic analysis between the MnBPDF and MnBPD groups revealed that the MnBPDF group upregulated oxidative stress-related pathways and antigen processing/presentation pathways (Fig. 5I and J), indicating enhanced oxidative stress induction in tumor cells and activation of DCs of the MnBPDF group.

### In vivo therapeutic efficacy of MnBPDF against orthotopic GBM

**Fig. 6:**
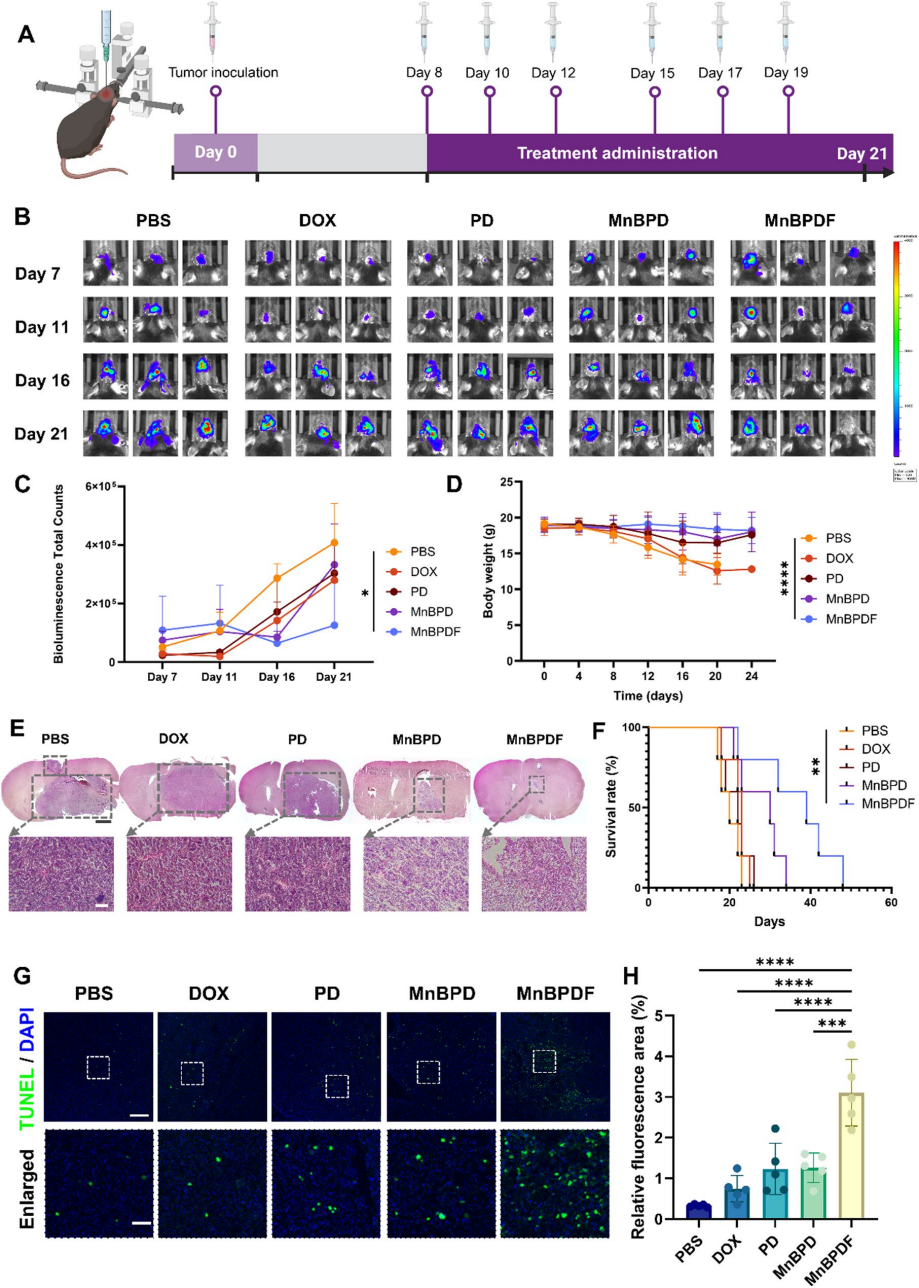
In Vivo Therapeutic Efficacy of MnBPDF in Orthotopic GBM Models. (**A**) Schematic of drug injection strategy for intracranial GBM. (**B**) Representative bioluminescence images of orthotopic GL261-Luc tumors at days 7, 11, 16 and 21 in different treatment groups. (**C**) Quantification of tumor bioluminescence intensity (*n* = 3). (**D**) Body weight dynamics (*n* = 5). (**E**) H&E-stained tumor sections after treatment on day 21. (Scale bars: 500 μm overview; 50 μm enlarged view). (**F**) Kaplan-Meier survival curves in each treatment group (*n* = 5). (**G**) TUNEL staining of apoptotic tumor cells in mice brain tumor area after treatment on day 21. (Scale bars: 200 μm overview; 40 μm enlarged view). (**H**) Semi-quantification of TUNEL fluorescence signals (*n* = 5)

Orthotopic GL261 GBM model in C57BL/6 mice were established to evaluate the antitumor efficacy of MnBPDF (Fig. 6A). Tumor-bearing mice were intravenously administered with 250 µL formulations (DOX, PD, MnBPD, or MnBPDF via tail vein injection on post-implantation days 8, 10, 12, 15, 17, and 19 (4 mg/kg DOX cumulative dose).

Bioluminescence imaging of GL261-Luc tumors revealed that MnBPDF treatment effectively suppressed intracranial tumor progression after six-dose administration (Fig. 6B and C). Brain sections H&E staining results on day 21 demonstrated a remarkable reduction in tumor volume within the MnBPDF group compared to other formulations (Fig. 6E).

The therapeutic benefits were further corroborated by longitudinal monitoring of body weight and survival outcomes. In contrast to the PBS and DOX groups, where progressive GBM led to noticeable body weight loss, MnBPDF-treated mice maintained stable body weight throughout the treatment period (Fig. 6D) [44]. Furthermore, the MnBPDF group exhibited significantly prolonged survival, with a median survival of 39 days compared to 20 days in the PBS group (Fig. 6F), highlighting both effective tumor suppression and favorable systemic tolerability.

**Fig. 7:**
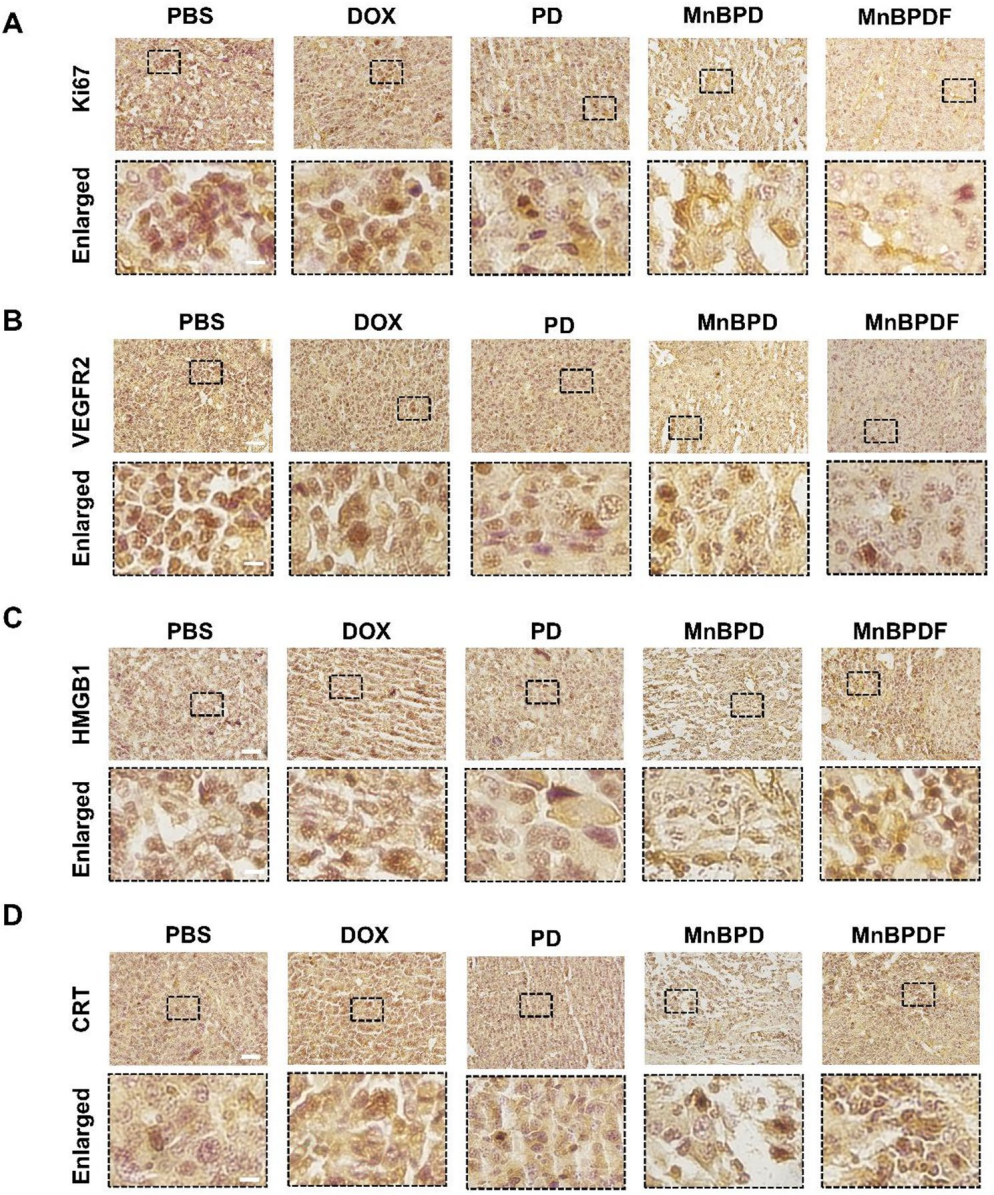
IHC Profiling of Orthotopic GBM on day 21. (**A**) Ki67 IHC staining of the mouse brain tumor area. (**B**) VEGFR2 IHC staining of the tumor area. (**C**) HMGB1 secretion IHC staining of the tumor area. (**D**) CRT surface exposure in the tumor area. (Scale bars: 50 μm overview; 10 μm enlarged view)

Mechanistic investigations through histopathological analysis unveiled multifaceted antitumor mechanisms. TUNEL staining demonstrated the highest level of apoptosis in MnBPDF-treated tumors, showing a 9.6-fold increase compared to PBS controls (Fig. 6G and H). The Ki67 IHC staining revealed remarkably suppressed proliferative activity in the MnBPDF-treated group compared to the control (Fig. 7A). Furthermore, VEGFR2 staining indicated potent anti-angiogenic effects, relative to other treatment groups (Fig. 7B).

Immunohistochemical analysis of ICD markers in mice brain tumor tissue after treatment revealed markedly upregulated surface exposure of HMGB1 and CRT in MnBPDF-treated tumors (Fig. 7C and D). PBS and DOX treatment groups exhibited a detectable level of HMGB1 positivity. It is plausible that this observation results from the inherent rapid growth of tumors, which can lead to inadequate nutrient supply and subsequent necrosis of tumor tissue, resulting in passive HMGB1 release [45].

### MnBPDF enhances DCs maturation and T cell infiltration in TME

**Fig. 8:**
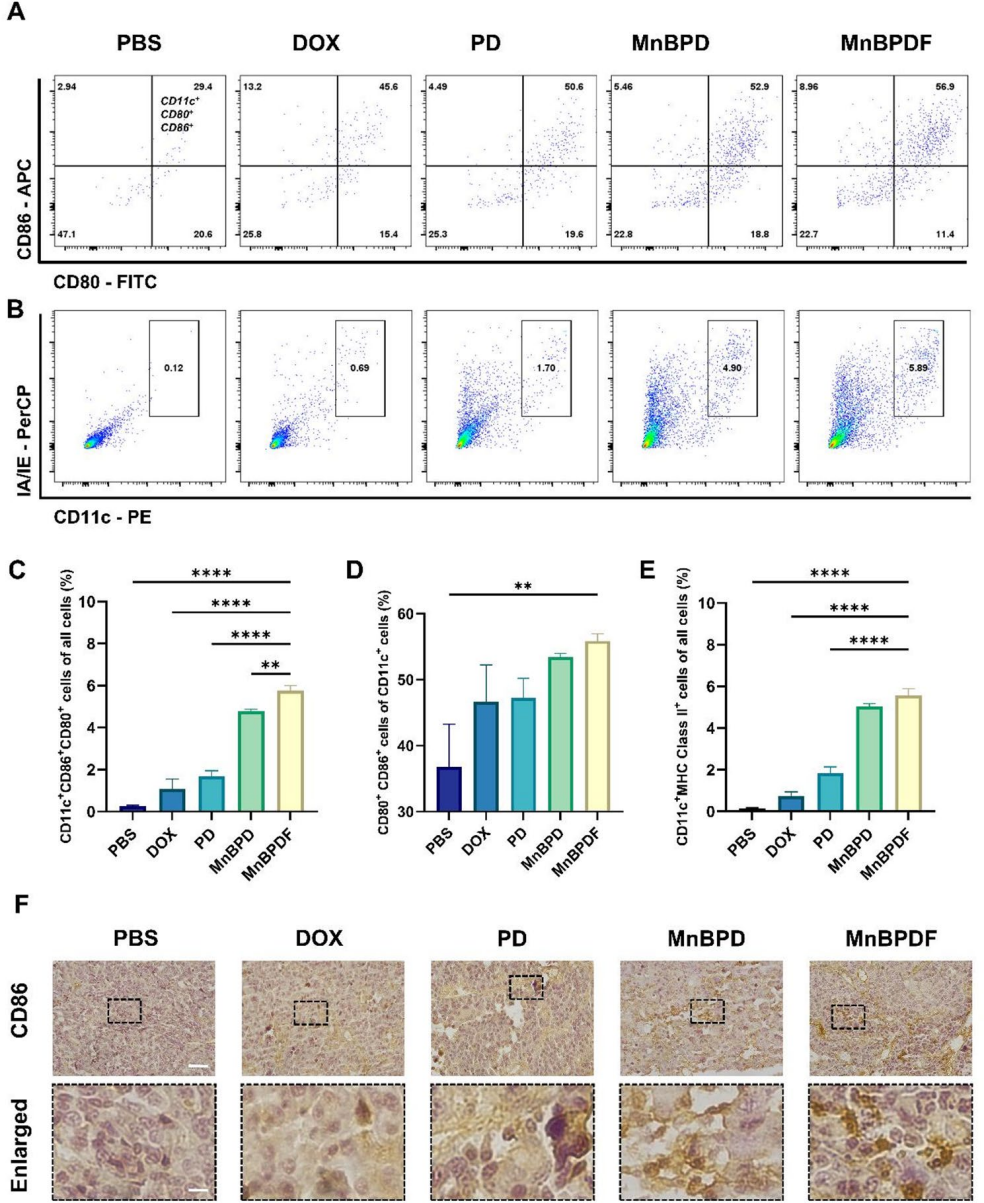
MnBPDF improves DCs Maturation in Vivo. (**A-B**) Flow cytometric quantification of tumor-infiltrating DCs maturation markers on day 21. CD11c^+^CD80^+^CD86^+^ (**A**), CD11c^+^MHCII^+^ (**B**). (**C**) Statistical analysis of CD11c^+^CD80^+^CD86^+^ cells of all cells in tumor tissue. (**D**) Statistical analysis of CD80^+^CD86^+^ cells of CD11c^+^ cells. (**E**) Statistical analysis of CD11c^+^MHCII^+^ cells of all cells in tumor tissue. (**F**) IHC staining of CD86 expression in tumor sections. (Scale bars: 50 μm overview; 10 μm enlarged view)

To investigate the immunomodulatory effects of MnBPDF on TME, we systematically assessed DCs maturation and T lymphocyte infiltration in orthotopic GBM-bearing mice after 6-dose treatment. Flow cytometric analysis revealed substantial enhancement of DCs maturation and adaptive immune activation in orthotopic GBM models in the MnBPDF-treated group. The proportion of CD11c^+^CD80^+^CD86^+^ DCs in the MnBPDF group (5.77 ± 0.24%) was significantly higher compared to PBS (0.27 ± 0.04%), DOX (1.07 ± 0.48%), PD (1.68 ± 0.26%), and MnBPD group (4.77 ± 0.10%) (Fig. 8A and C). CD11c^+^MHCII^+^ DCs in the MnBPDF group reached 5.58 ± 0.30%, demonstrating a significant increase in activation over PBS (0.13 ± 0.03%), DOX (0.73 ± 0.21%), and PD (1.83 ± 0.31%) groups (Fig. 8B and E). Within the CD11c⁺ compartment, MnBPDF induced a significant elevation in CD80⁺CD86⁺ subsets (55.84 ± 1.11%) compared to PBS (36.77 ± 6.47%, Fig. 8D). IHC Staining of CD86 in mouse brain tumor tissue sections revealed a significantly higher number of CD86^+^ cells in the MnBPDF group compared to the other treatment groups (Fig. 8F). These results indicate that treatment with MnBPDF leads to a higher proportion of mature DCs within the mouse brain tumor tissue.

**Fig. 9:**
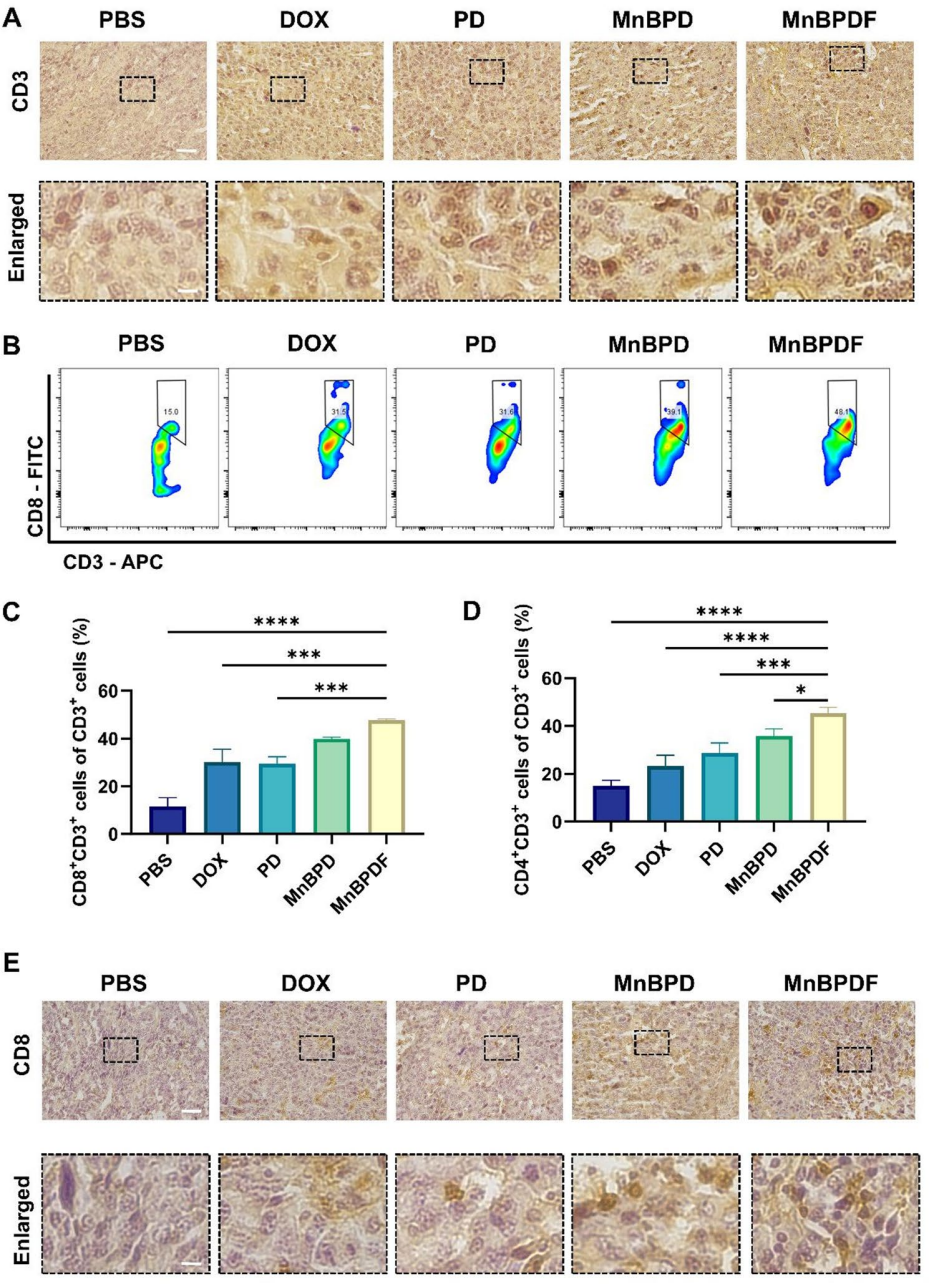
MnBPDF Promotes CTLs Infiltration and Enhances T Cell Immunity. (**A**) IHC staining of CD3 expression in tumor area. (Scale bars: 50 μm overview; 10 μm enlarged view). (**B**) Flow cytometry analysis of tumor-infiltrating CD3⁺CD8⁺ T cells on day 21. (**C**) Statistical analysis of CD3⁺CD8⁺ T cells percentage in CD3^+^ T cells in tumor. (**D**) Statistical analysis of CD3⁺CD4⁺ T cells percentage in CD3^+^ T cells in tumor. (**E**) IHC staining of CD8 expression. (Scale bars: 50 μm overview; 10 μm enlarged view)

The effective presentation of tumor-associated antigens to T cells by mature DCs constitutes a pivotal mechanism in adaptive antitumor immunity [46]. IHC staining of mice tumors revealed an elevation in infiltrating CD3^+^ T cells in the TME of the MnBPDF group (Fig. 9A). The proportion of infiltrating CD8^+^ T cells and CD4^+^ T cells in the MnBPDF group was significantly higher compared to the other treatment groups (Fig. 9C and D). Notably, the proportion of infiltrating CD8^+^ T cells in the MnBPDF group was significantly elevated to 47.7 ± 0.47%, approximately 4.1-fold higher than PBS group (Fig. 9B). IHC staining also demonstrated an increase in infiltrating CD8^+^ T cells in the TME of the MnBPDF group (Fig. 9E). This coordinated expansion of both CD4⁺ and CD8⁺ cells align with the function of mature DCs in MHC I/II-mediated antigen cross-presentation.

### Biosafety evaluation of MnBPDF

**Fig. 10:**
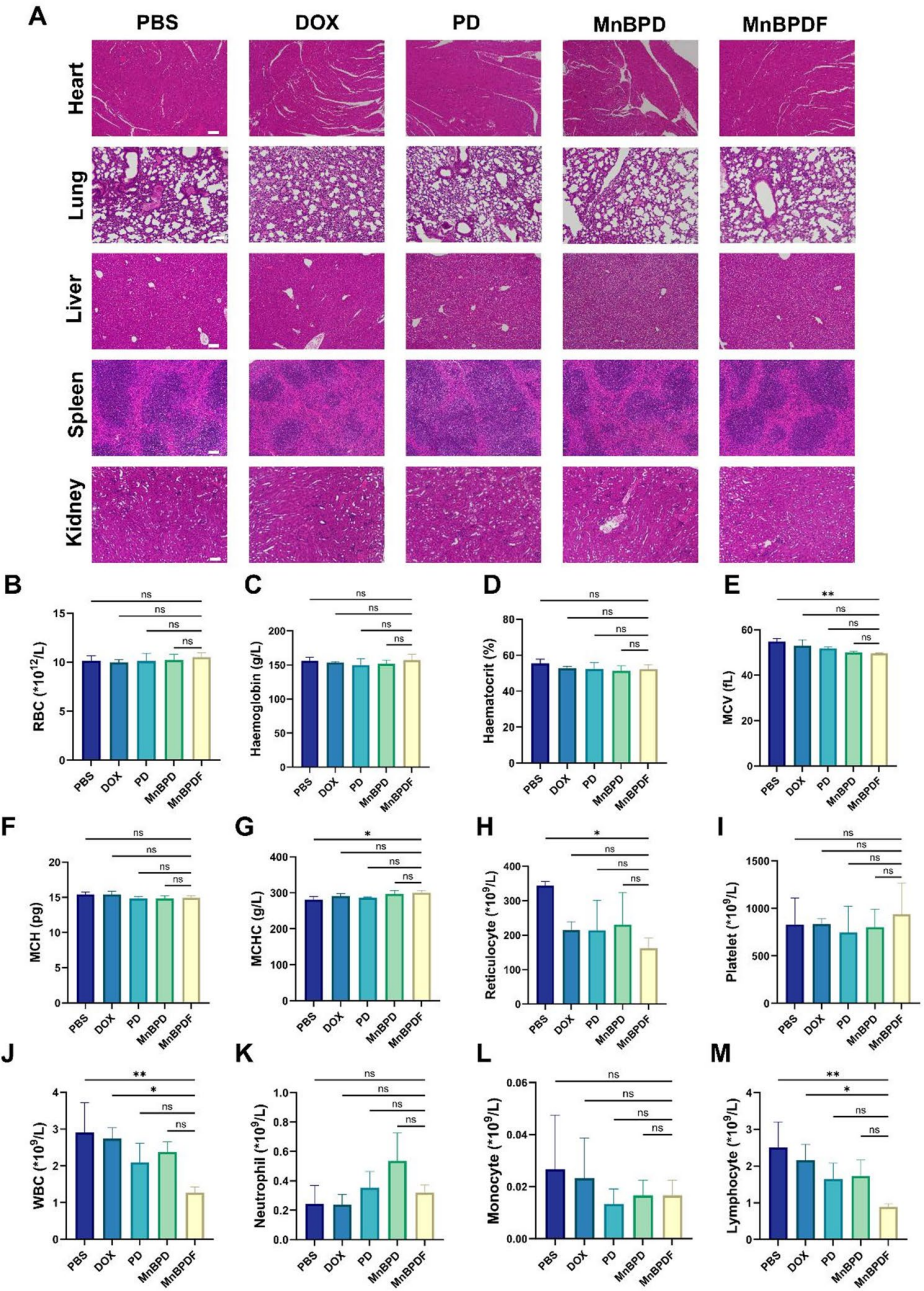
Biosafety Evaluation of MnBPDF. (**A**) H&E staining of major organs. Scale bar = 100 μm. (**B**–**L**) CBC after treatment with PBS, DOX, PD, MnBPD, MnBPDF on day 21. RBC (**B**), Haemoglobin (**C**), Haematocrit (**D**), MCV (**E**), MCH (**F**), MCHC (**G**), Reticulocyte Count (**H**), Platelet (**I**). WBC **(J)**, Neutrophil Count (**K**), Monocyte Count (**L**), Lymphocyte Count (**M**). (n = 3)

To evaluate the systemic safety profile of MnBPDF, we conducted comprehensive histopathological and hematological analyses following 6-dose administration. Major organ histopathological analysis showed no discernible pathological alterations across all treatment groups, confirming the absence of MnBPDF-induced organ toxicity (Fig. 10A).

Complete blood count (CBC) analysis confirmed the exceptional erythrocyte compatibility of MnBPDF. Hematologic profiling remained stable across treatment groups, with no statistically relevant deviations observed in these erythrocyte indices (Fig. 10B-D). Similarly, cellular volumetric parameters showed values within normal physiological ranges (Fig. 10E-G). Reticulocyte count and platelet levels also exhibited no treatment-related fluctuations, further substantiating the hematological safety profile of MnBPDF (Fig. 10H-I). The MnBPDF-treated group demonstrated a modest reduction in total WBC counts relative to PBS and DOX groups, which were primarily attributable to decreased lymphocyte populations as neutrophil and monocyte counts maintained comparable levels across all experimental groups (Fig. 10J-M). This peripheral lymphopenia reflects active lymphocyte recruitment to tumor sites, as evidenced by the 4.8-fold increase in tumor-infiltrating T cells (Fig. 9A-D), consistent with previous reports on immunotherapy-induced immune cell trafficking [47].

## Conclusion

In summary, we engineered a multifunctional nanoassembly, MnBPDF, integrating Mn^2+^ chelated BCD coating with PLGA-encapsulated DOX and Fe₃O₄ nanoparticles for targeted GBM therapy. DOX induces ICD, releasing DAMPs, while Fe²⁺/Mn²⁺ ions amplify oxidative stress via Fenton-like catalysis. Tumor-derived dsDNA activates the cGAS-STING pathway in APCs, with Mn²⁺ acting as an effective STING agonist, which induces the secretion of IFN-I and enhances the activation of CTLs. By synergistically integrating chemotherapy, metalloimmunology, and redox modulation, this work establishes a multimodal nano-immunotherapeutic platform that achieves precision-targeted GBM therapy.

Future research should also explore strategies to enhance BBB penetration efficiency and enable real-time monitoring of therapeutic response using integrated imaging modalities. In addition, further mechanistic studies are warranted to elucidate the regulation of the tumor immune microenvironment and the dynamic expression patterns of immune-related cytokines throughout the treatment process. Given that key components of MnBPDF, including Fe₃O₄ and manganese-based agents, have been approved by the FDA for clinical use, this platform holds translational potential for further development as a clinically applicable nanotherapeutic strategy for GBM.

## Supplementary Information

Supplementary Material 1.

## Data Availability

The data that supports the findings of this study are available from the corresponding author upon reasonable request..
