# Supplementary material for: Manganese ion chelated nanoassemblies synergizing metalloimmunotherapy - chemodynamic for potentiating glioblastoma treatment
Source: J Nanobiotechnology. 2025 Dec 19;23:775. doi: 10.1186/s12951-025-03845-6 (PMC12717771; doi:10.1186/s12951-025-03845-6)
Supplement: Supplementary file 1 — Supplementary Material 1. [file 12951_2025_3845_MOESM1_ESM.docx]

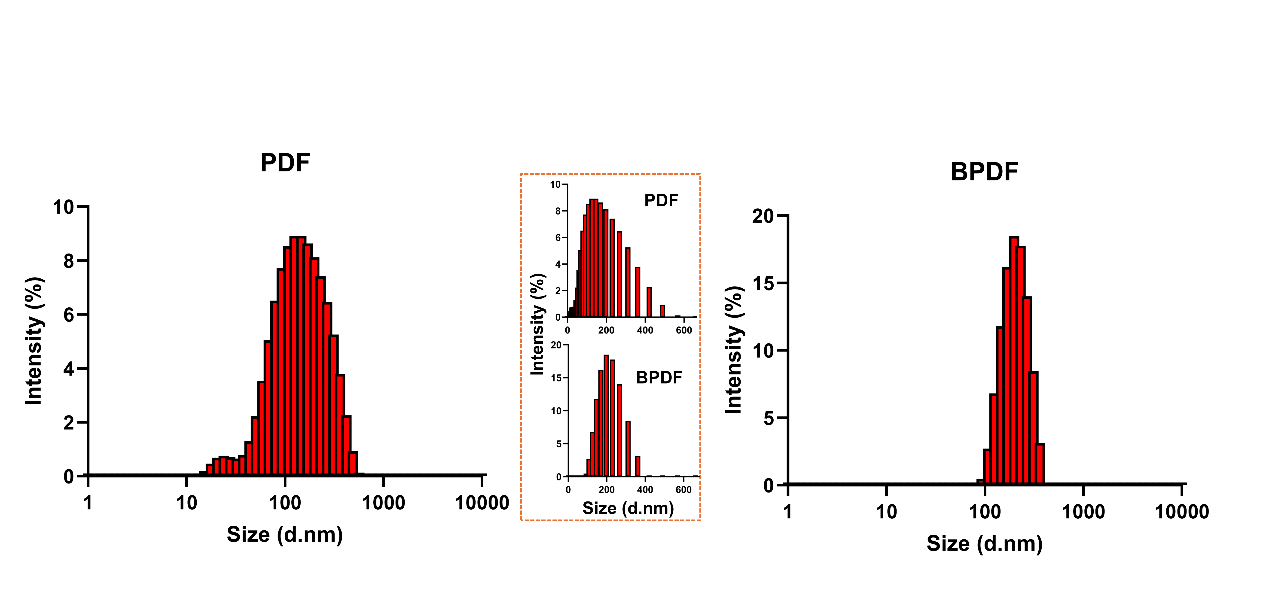


**Figure. S1** Hydrodynamic diameter distribution of PDF and BPDF measured by DLS.


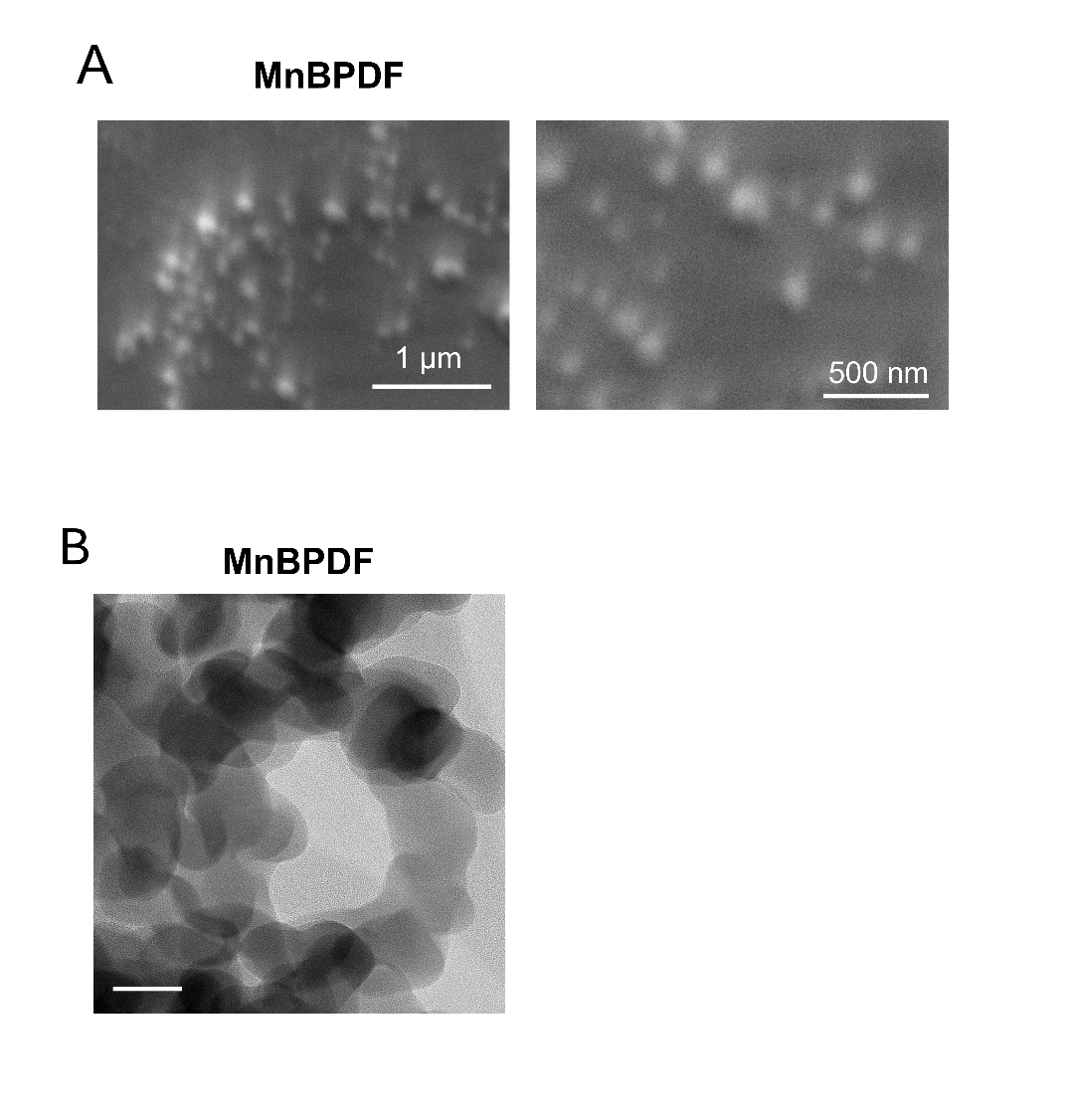


**Figure. S2** (A) SEM image of MnBPDF. (B) TEM image of MnBPDF. Scale bar = 100 nm.

**
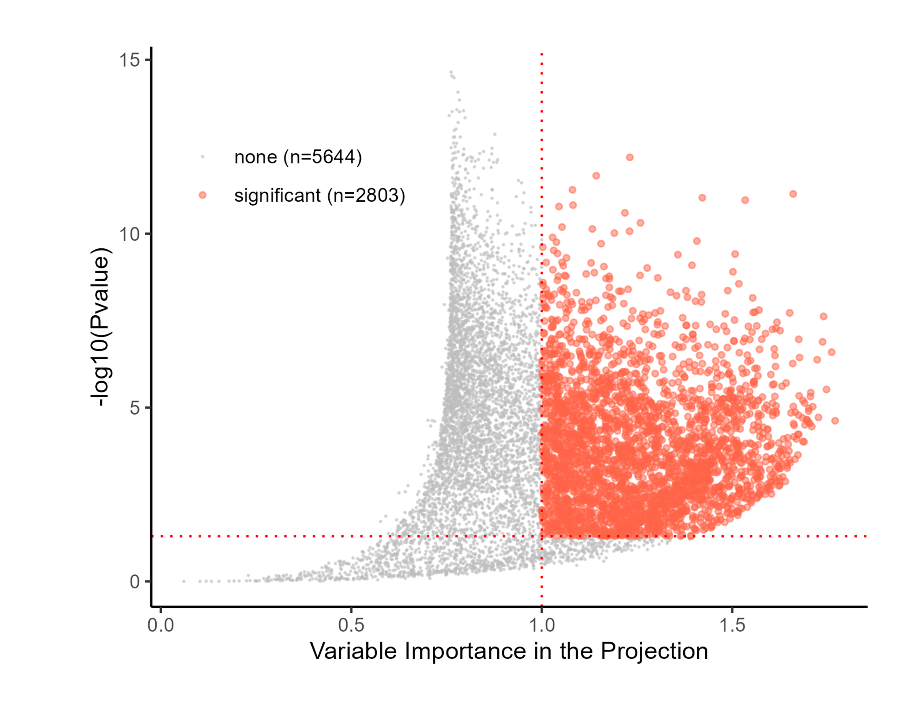
**

**Figure. S3** Differential protein expression analysis (ANOVA, p < 0.05; VIP > 1) identified 2,803 significantly altered proteins (of 8,447 quantified) across multiple treatment groups (PBS, PDF, MnBPD, MnBPDF) using proteomics.


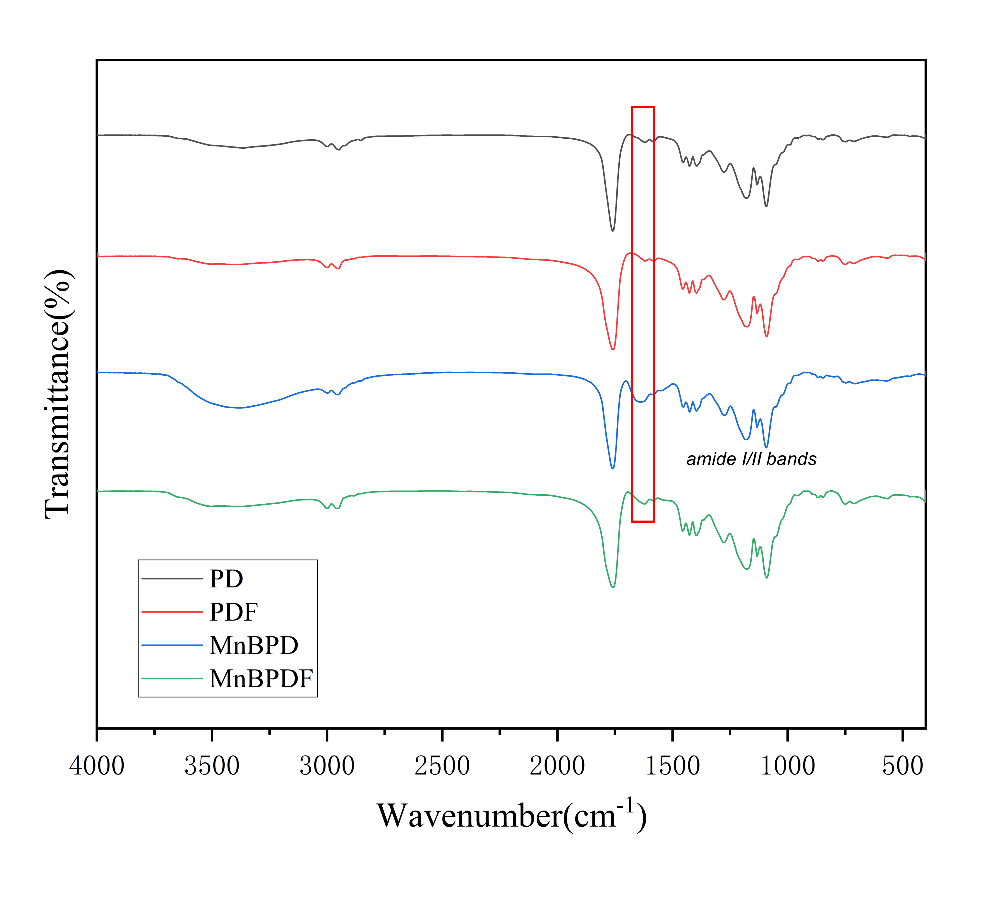


**Figure. S4** FTIR spectra of PD, PDF, MnBPD, and MnBPDF

**
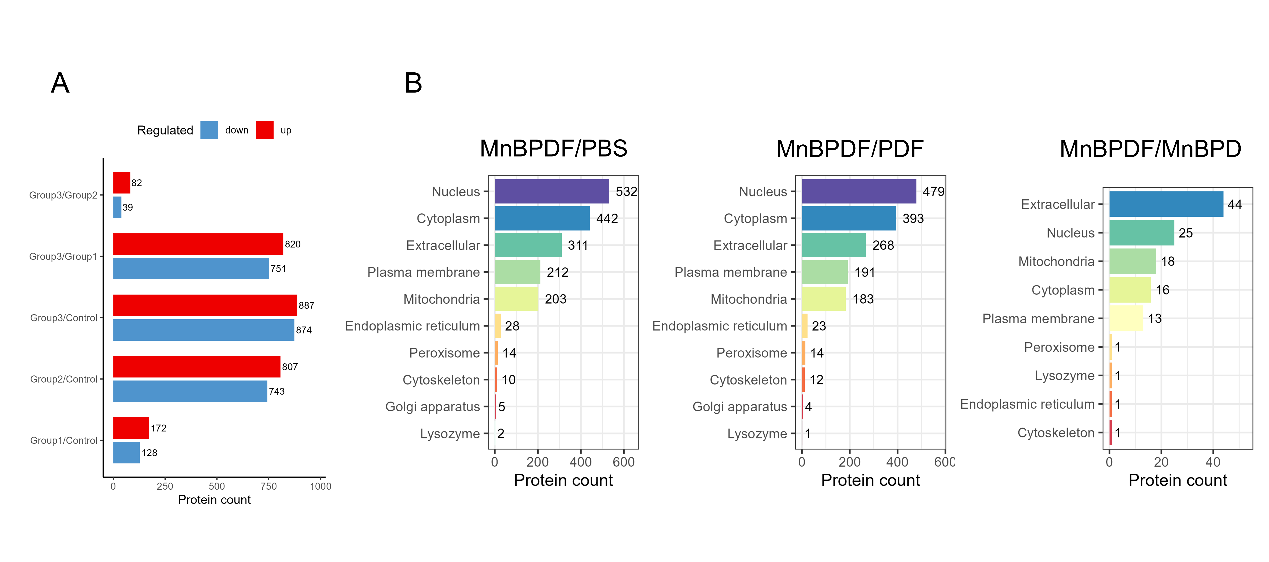
**

**Figure. S5** (A) Statistical analysis of proteomic differences between groups (|Fold change| > 1.5). (B) Subcellular localization analysis of differential functions between the MnBPDF group and other treatment groups.


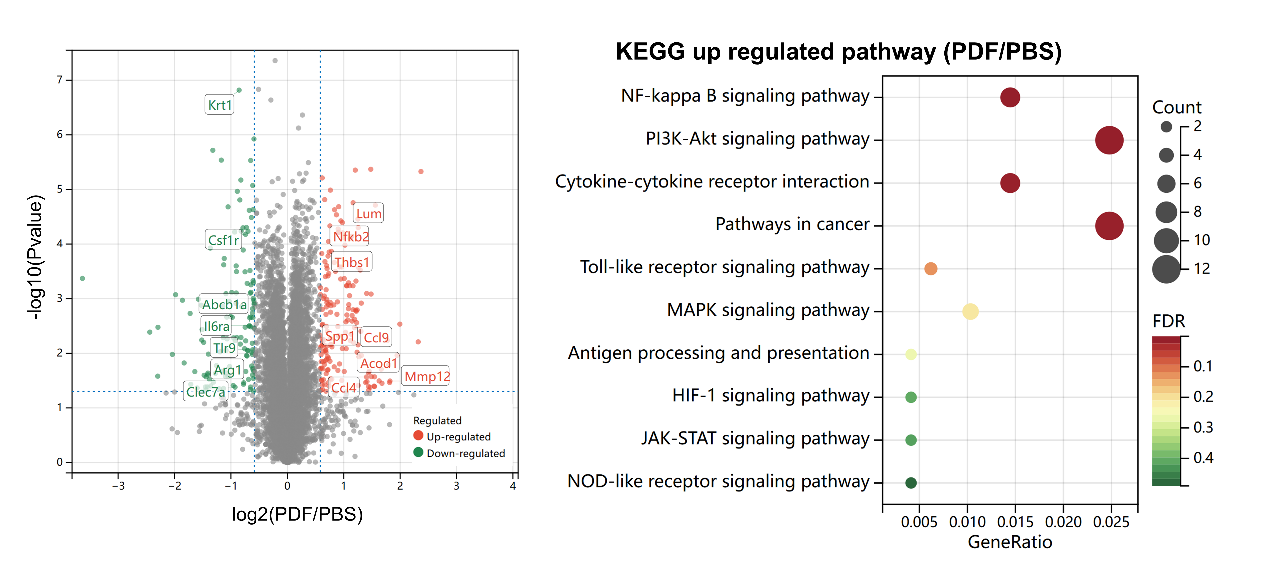


**Figure. S6** (A) Volcano plot analysis between PDF and PBS group (|fold change| >1.5, p<0.05). (B) KEGG pathway enrichment analysis of upregulated pathways between PDF group and PBS group. (FDR < 0.05, p < 0.05).


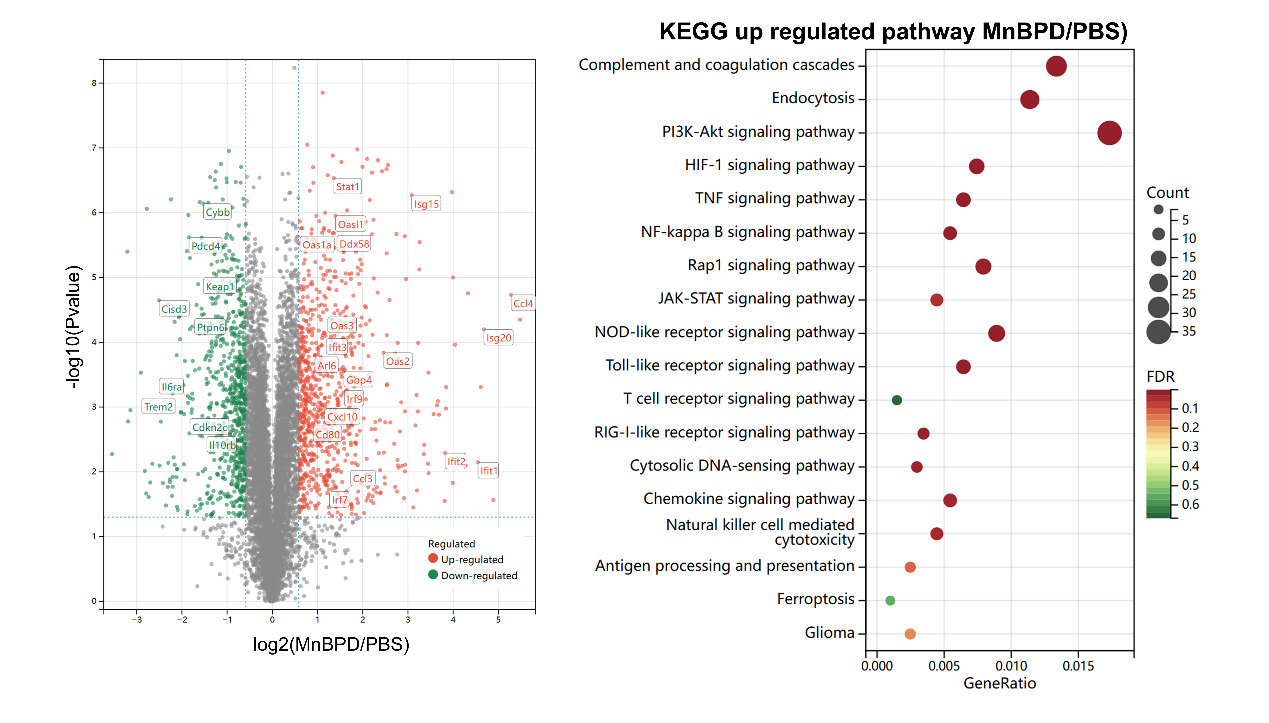


**Figure. S7** (A) Volcano plot analysis between MnBPD and PBS group (|fold change| >1.5, p<0.05). (B) KEGG pathway enrichment analysis of upregulated pathways between PDF group and PBS group. (FDR < 0.05, p < 0.05).


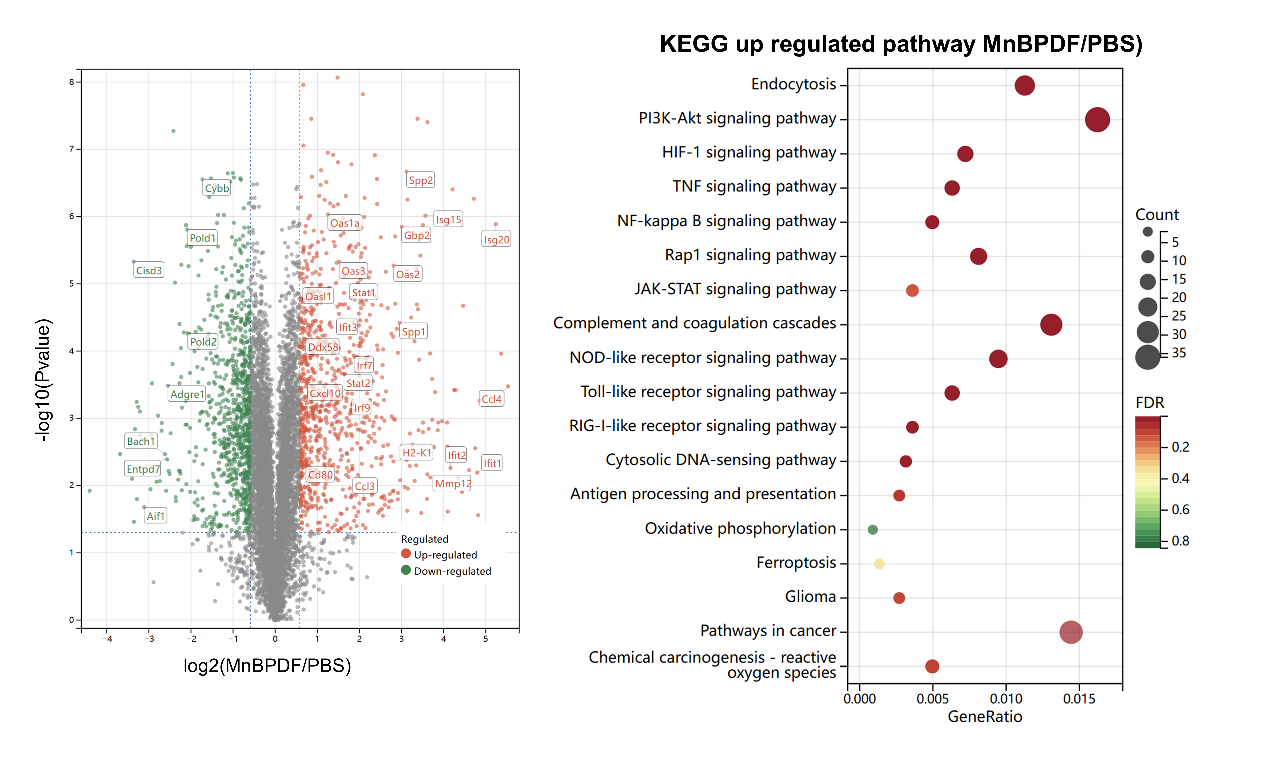


**Figure. S8** (A) Volcano plot analysis between MnBPDF and PBS group (|fold change| >1.5, p<0.05). (B) KEGG pathway enrichment analysis of upregulated pathways between PDF group and PBS group. (FDR < 0.05, p < 0.05).
